# Drivers of food consumption among overweight mother-child dyads in Malawi

**DOI:** 10.1371/journal.pone.0243721

**Published:** 2020-12-17

**Authors:** Chrissie Thakwalakwa, Valerie L. Flax, John C. Phuka, Harrison Garcia, Lindsay M. Jaacks

**Affiliations:** 1 Centre for Social Research, Chancellor College, University of Malawi, Zomba, Malawi; 2 RTI International, Research Triangle Park, North Carolina, United States of America; 3 College of Medicine, University of Malawi, Blantyre, Malawi; 4 Department of Global Health and Population, Harvard T.H. Chan School of Public Health, Boston, Massachusetts, United States of America; San Raffaele Roma Open University, ITALY

## Abstract

To address the increase in overweight and obesity among mothers and children in sub-Saharan Africa, an understanding of the factors that drive their food consumption is needed. We hypothesized food consumption in Malawi is driven by a combination of factors, including season, food accessibility (area of residence, convenience of purchasing food, female autonomy), food affordability (household resources, food expenditures, household food insecurity), food desirability (taste preferences, body size preferences), demographics, and morbidity. Participants in Lilongwe and Kasungu Districts were enrolled across three types of mother-child dyads: either the mother (n = 120), child (n = 80), or both (n = 74) were overweight. Seven-day dietary intake was assessed using a quantitative food frequency questionnaire during the dry and rainy seasons. Drivers associated with intake of calories, macronutrients, and 11 food groups at p<0.1 in univariate models were entered into separate multivariate linear regression models for each dietary intake outcome. Mother-child dyads with an overweight child had a higher percent of calories from carbohydrates and lower percent of calories from fat compared to dyads with a normal weight child (both p<0.01). These mothers also had the highest intake of grains (p<0.01) and their children had the lowest intake of oil/fat (p = 0.01). Household food insecurity, maternal taste preferences, and maternal body size preferences were the most consistent predictors of food group consumption. Household food insecurity was associated with lower intake of grains, fruits, meat and eggs, oil/fat, and snacks. Maternal taste preferences predicted increased consumption of grains, legumes/nuts, vegetables, fish, and oil/fat. Maternal body size preferences for herself and her child were associated with consumption of grains, legumes/nuts, dairy, and sweets. Predictors of food consumption varied by season, across food groups, and for mothers and children. In conclusion, indicators of food affordability and desirability were the most common predictors of food consumption among overweight mother-child dyads in Malawi.

## Introduction

The prevalence of overweight and obesity in sub-Saharan Africa (SSA) is rapidly increasing. Half of the population is predicted to be overweight or have obesity by 2030 [1]. Malawi, one of the poorest countries in SSA, is no exception. The prevalence of overweight and obesity among women of reproductive age in Malawi increased from 10% in 1992 to 21% in 2015–16 [2]. Indeed, overweight and obesity are now more common than underweight among women (21% overweight or have obesity versus 7% underweight), and more common than wasting among children under five (5% overweight versus 3% wasted [thin for age]) [2]. This rapid increase in overweight and obesity in Malawi and other countries in SSA [1], which has not been accompanied by a similarly rapid decline in undernutrition [3], has resulted in these countries carrying a significant double burden of malnutrition [4]. Even within the same household, discrepancies in the nutritional status of children and women frequently exist [5].

Rapidly changing food systems in SSA likely underlie this transition [4]. However, there is currently no data from low-income SSA countries on the factors driving food consumption in households where the mother, the child, or both are overweight. Information on dietary intake in Malawi, in general, is lacking. A handful of previous studies in rural areas focused on undernutrition showed that dietary intakes of Malawian children are inadequate in terms of dietary quantity and quality [6–10]. After a review of literature, only one paper on maternal diet in Malawi was found, which focused on pregnant HIV-infected women and reported that dietary patterns differed by socioeconomic status [11].

We hypothesized that decisions on food consumption in Malawi may be driven by a combination of factors, including season, area of residence, convenience of purchasing food, demographics, household resources, expenditures on food, household food security, cultural beliefs about body size, female autonomy, taste preferences, and maternal and child morbidity. These drivers can be mapped onto the recently proposed conceptual framework of the Agriculture, Nutrition and Health Academy Food Environment Working Group (ANH-FEWG) [12]. The ANH-FEWG framework conceptualizes the food environment as the interface between an individual and the wider food system, consisting of two domains: (1) the external domain, which includes, for example, food availability, prices, and vendor properties, and (2) the personal domain, which includes food accessibility, affordability, convenience, and desirability [12]. In this study, we focused on factors in the personal domain and on individual and household characteristics that influence the choice of foods consumed by women and children in households experiencing the nutrition transition. We considered the external domain by analyzing food consumption during the rainy and dry seasons, when food availability and prices vary. Our specific study objectives were to compare the dietary intake of mothers and children across three types of mother-child dyads: (1) overweight mothers with an overweight child, (2) overweight mothers with a normal weight child, and (3) normal weight mothers with an overweight child, and to explore a comprehensive set of predictors to understand drivers of food consumption in this population.

## Materials and methods

### Sample population

The study was conducted in Lilongwe and Kasungu Districts in the Central Region of Malawi, which had the highest prevalence of overweight among women and children in the most recent Demographic and Health Survey [2]. The data collection took place in both the dry season (May-October 2017 and 2018) and rainy season (January-April 2018 and 2019), on average, 6.6 ±1.0 months (range: 4.1 to 10.7 months) later. In each district, data were collected in two urban neighborhoods and two rural villages. Local leaders in the study sites invited all women with children less than 5 years for anthropometric screening to determine study eligibility.

Standing height of mothers and children ≥2 years was measured to the nearest 0.1 cm using a portable stadiometer (Seca 213). Recumbent length of children <2 years was measured to the nearest 0.1 cm using an infant measuring mat (Seca 210). Weight of mothers and children was measured to the nearest 0.1 kg using a digital scale (Seca 803 for mothers and children ≥2 years; Seca 354 for children <2 years). Three types of mother-child dyads were purposefully enrolled based on their nutritional status as determined during the screening: (1) overweight mothers (body mass index [BMI] ≥25 kg/m^2^) with an overweight child (weight-for-height z-score [WHZ] >+2 SD), (2) overweight mothers with a normal weight child (WHZ> -2 SD and WHZ ≤+2 SD), and (3) normal weight mothers (BMI ≥18 kg/m^2^ and BMI <25 kg/m^2^) with an overweight child. The sample sizes for the mother-child dyads were as follows: overweight mother, overweight child (n = 74); overweight mother, normal weight child (n = 120); and normal weight mother, overweight child (n = 80). Additional eligibility criteria included: mothers aged ≥18 years, children aged 6–59 months, and mothers were the biological parent of the enrolled child. Data on pregnancy status were not collected.

### Ethics approval and consent to participate

The study was approved by the College of Medicine Research Ethics Committee at the University of Malawi and by the institutional review boards at RTI International and the Harvard T.H. Chan School of Public Health. Participants provided signed informed consent. They received an incentive equivalent to $4 USD.

### Diet assessment

All data were collected by trained research assistants in the participants’ local language (Chichewa). Quantitative food frequency questionnaires (QFFQs) adapted from a previous study in South Africa [13–15] were used to assess habitual dietary intake of mothers and children. The QFFQ included food flash cards (photos) for all foods based on lists from existing Malawi food composition tables [10, 16] and scans of food items available at local stores and markets. Data were collected on diet recalled for the previous 7 days. The mother was asked to create one pile of food cards showing items she rarely (once every few months) or never ate or drank and then to divide the remaining cards into items she ate or drank occasionally (once or twice a month) and those she ate regularly (every day or every week) [17]. She was then asked for information on the frequency and amounts of the regular food items, repeating the same process for the child. Intake of cooking oil or butter/margarine of 50 grams per day or more was divided by household size as research assistants indicated that these values were reported at the level of the household rather than the individual.

Eleven food groups were derived from the data to evaluate as outcomes, separately for mothers and children: grains, roots/tubers, vegetables, fruits, meat/eggs, fish, dairy, legumes and nuts, oil/fat, snacks, and sweets (S1 Table). Total energy (kcal/day) and percent of energy from carbohydrates, fat, and protein were determined using a Malawi food composition table [18]. Nutrient composition for 37 missing food items was obtained from the Tanzania food composition table [19] or the USDA food composition database [20].

### Drivers of food consumption

Drivers of food consumption specified *a priori* and their link to the food environment conceptual framework are summarized in Table 1 [12]. We also included several other individual and household level factors that have been identified in previous studies of drivers of dietary behaviors in SSA [21].

**Table 1:** Summary of drivers of food consumption evaluated in this study of overweight mother-child dyads in Malawi.

Food environment category	Food environment sub-categories	Variables
Accessibility	Physical distance, mode of transport, autonomy	Residence in Lilongwe versus Kasungu, urban versus rural residence, how woman gets to nearest market/shop to purchase food, how long it takes to get to nearest market/shop to purchase food, who purchases most food, female autonomy
Affordability	Purchasing power	Household food expenditures, amount spent on special foods for children <5 years Proxies for purchasing power: main source of drinking water, toilet facility used by the household, total number of household assets, household food insecurity
Desirability	Preferences, tastes, desires, attitudes, culture	Maternal taste preferences, mother’s body size preference for herself and her child, mother’s perception of a healthy body size for herself and her child, purchasing special foods for children <5 years in household
Other factors	Demographic	Age of mother, age of child, child’s sex, maternal educational attainment, household size, number of children <5 years in the household
	Morbidity	Maternal and child morbidity (diarrhea, fever, and cough)

#### Accessibility

Access to food relates to physical location, distance to food retailers, mode of transport, and who has the responsibility to purchase food for the household. Specific variables considered in this category were residence in Lilongwe (referent) versus Kasungu, urban (referent) versus rural residence, how the woman gets to the nearest market/shop to purchase food (walk [referent] or other), and how long it takes to get to nearest market/shop to purchase food (minutes).

We also included who purchases most food for the household (mother [referent], husband/partner, both, or other family member) and female autonomy score in the food access category. A validated multidimensional construct was used to measure female autonomy. Mothers were asked eight questions on freedom from violence, participation in non-economic family decisions, community involvement, and participation in household economic decisions, with a score of one being recorded for each question where decisions were made herself or jointly with a husband or partner while a score of zero was assigned in all other cases [22]. For the final question, which asked if respondents thought a husband is justified in hitting or beating his wife in each of five situations, 0.2 points were given for each time that the mother reported that the husband was not justified. Points given for all questions were then summed to create the female autonomy score, with a higher score indicating higher autonomy.

#### Affordability

A questionnaire adapted from a Feed the Future evaluation in Malawi was used to determine household assets and expenditures on food [23]. Food expenditure was recalled over the past 7 days and the total amount spent on food for the household in a typical week was calculated in local currency and converted to USD using the World Bank official exchange rate for 2018 of 732.33 (accessed October 1, 2019). This was also how total amount spent on special foods for children <5 years in the household was calculated (USD converted using World Bank official exchange rate for 2018 of 732.33 [accessed October 1, 2019]).

Proxies for purchasing power included main source of drinking water (piped [referent], well, or borehole), toilet facility used by the household (flush [referent], ventilated improved latrine, pit latrine with roof, or traditional pit latrine/no facility), total number of household assets, and household food insecurity. A household asset score was calculated as the total number of the following assets owned by the household (maximum score of 12): electricity, koloboyi (home-made kerosene lamp), paraffin lamp, radio, television, mattress, sofa set, table and chair(s), refrigerator, watch, bicycle, and mobile telephone [2]. The Household Food Insecurity Access Scale (HFIAS) questionnaire was used to assess household food insecurity. Participants were asked to recall how frequently over the past four weeks each of nine conditions was experienced, covering the following three domains: (i) anxiety and uncertainty, (ii) insufficient quality, and (iii) insufficient food intake and its physical consequences [24]. Responses were used to calculate an HFIAS score with a minimum of 0, if the household responded “no” to the occurrence of all conditions, and a maximum of 27, if the household responded “yes” to the occurrence of all conditions with a frequency of “often” for all conditions. Thus, higher HFIAS scores corresponded to greater food insecurity [24].

#### Desirability

This category included variables related to preferences, tastes, desires, attitudes, and culture. Specific variables included maternal taste preference for the corresponding food group (e.g., preference for grains as a predictor of grain intake), mother’s body size preference for herself and her child (underweight [referent], normal weight, overweight, or obese), mother’s perception of a healthy body size for herself and her child (underweight [referent], normal weight, overweight, or obese), and purchasing special foods for children <5 years in household (yes or no [referent]).

To assess maternal taste preferences, we used a taste preference checklist matching items in the QFFQ and representing all major foods consumed in this population [9, 10, 16]. Participants indicated how much they liked or disliked each item using a 5-point hedonic preference scale, ranging from “1 = extremely dislike” to “5 = extremely like” with “3 = neither like nor dislike” [25, 26]. Preferences for the 11 food groups were calculated as the arithmetic average preference for all foods in that group.

To assess body size preferences, a set of seven adult female and seven child body silhouette drawings were used. A local artist adapted mothers’ body silhouettes from versions previously used in Malawi [27–29] and adapted child body silhouettes from Hager et al. [29]. Each body silhouette drawing was printed separately on cardstock and laminated. The interviewer mixed the body silhouettes and laid them out in a random order before asking the mother to make selections for herself and then again for her child [30].

#### Other factors—Demographics and morbidity

Demographic characteristics included the age of the mother (years), the age of the child (months), child sex male (referent) versus female, household size, number of children <5 years in the household, and maternal educational attainment less than secondary school (referent) versus any secondary school or higher.

We also considered maternal and child morbidity over the past two weeks (yes or no [referent] to diarrhea, fever, and cough) as a driver. Morbidity questions for children were from the Malawi Demographic and Health Survey and the same questions were used for mothers [31].

All drivers were surveyed in both seasons except for mother’s body size perceptions and preferences for herself and the child, which were measured only in the dry season. We did not repeat anthropometric measurements in the rainy season because the analysis was based on the body size of the mother-child dyads at enrollment.

### Statistical analysis

Descriptive statistics were used to summarize the hypothesized drivers and dietary intake. Differences across the three types of mother-child dyads were determined using chi-square tests and Kruskal-Wallis H tests (a non-parametric alternative to one-way analysis of variance). Differences between seasons were tested using exact tests of symmetry (which reduce to McNemar’s tests in the case of binary variables) [32] and Wilcoxon signed-rank tests (a non-parametric alternative to a paired t-test).

The relationship between each driver (independent variables) and each of the four nutrient variables (total calories and percent of calories from carbohydrates, fat, and protein) and 11 food groups (dependent variables) was evaluated using linear regression. Drivers associated with the outcome at p<0.1 in univariate linear regression models (e.g. models with one independent variable) were entered into a multivariate linear regression model (e.g. models with more than one independent variable; 15 separate models, one for each of the dietary intake outcomes) and relationships significant at p<0.05 were presented in the results. Continuous drivers were rescaled to have a mean of zero and a standard deviation of one to ease interpretation and comparisons across variables. All analyses were conducted using Stata v. 14.2 (StataCorp, College Station, Texas, US).

## Results

A comparison of demographic and socio-economic characteristics between mother-child dyads is presented in Table 2. Mothers were (mean ±SD) 27.8 ±6.0 years old (range: 18–45 years) and their index children were 27.0 ±15.3 months old (range: 6–59 months). Just over half of the index children were female (53.5%) and about 42% of mothers had any secondary school education or higher. Overweight mothers were older and overweight children were younger compared to normal weight mothers and children (p<0.001). Dyads with overweight mothers had larger household sizes (p = 0.03) and dyads with overweight children were more likely to be male children (p = 0.07) and to purchase special foods for children (p = 0.02). Dyads with overweight children were also more likely to have pit latrines with a roof (p = 0.03) and had fewer household assets (p = 0.006). In particular, households in which the mother was normal weight and the child was overweight had the greatest food insecurity (p = 0.001), spent the least amount on food (p = 0.02), and the child was more likely to have diarrhea in the last 2 weeks (p = 0.02). Mothers in dyads with a normal weight child were more likely to select the underweight or normal weight body silhouette as preferred (p = 0.03).

**Table 2:** Summary of demographic and socio–economic characteristics across mother–child dyads in Malawi at baseline (dry season).

	Total (n = 274)	Overweight Mother, Overweight Child (n = 74)	Overweight Mother, Normal Weight Child (n = 120)	Normal Weight Mother, Overweight Child (n = 80)	P-value^1^
Age of mother, *years*	27.8 ±6.0	28.9 ±6.0	28.9 ±5.9	25.3 ±5.5	<0.001
Age of child, *months*	27.0 ±15.3	25.2 ±14.4	32.8 ±15.4	20.0 ±12.5	<0.001
Child sex					
Male	46.7 (128)	50.0 (37)	39.2 (47)	55.0 (44)	0.07
Female	53.3 (146)	50.0 (37)	60.8 (73)	45.0 (36)	
Location					
Lilongwe	50.4 (138)	50.0 (37)	50.0 (60)	51.3 (41)	0.98
Kasungu	49.6 (136)	50.0 (37)	50.0 (60)	48.8 (39)	
Residence					
Urban	50.4 (138)	50.0 (37)	52.5 (63)	47.5 (38)	0.78
Rural	49.6 (136)	50.0 (37)	47.5 (57)	52.5 (42)	
Household size	5.1 ±1.7	5.3 ±1.6	5.2 ±1.8	4.7 ±1.6	0.03
Number of children <5 years in household	1.3 ±0.6	1.3 ±0.7	1.4 ±0.7	1.3 ±0.5	0.79
Maternal educational attainment					
<Secondary school	58.4 (160)	59.5 (44)	53.3 (64)	65.0 (52)	0.26
Any secondary school or higher	41.6 (114)	40.5 (30)	46.7 (56)	35.0 (28)	
Main source of drinking water					
Piped into dwelling, plot/yard, or communal standpipe	56.2 (154)	51.4 (38)	60.8 (73)	53.8 (43)	0.60
Well in yard/plot or public well	12.8 (35)	16.2 (12)	11.7 (14)	11.3 (9)	
Borehole	31.0 (85)	32.4 (24)	27.5 (33)	35.0 (28)	
Toilet facility used by household					
Flush toilet	5.8 (16)	5.4 (4)	8.3 (10)	2.5 (2)	0.03
Ventilated improved pit latrine	18.3 (50)	12.2 (9)	25.8 (31)	12.5 (10)	
Pit latrine with roof	61.3 (168)	62.2 (46)	54.2 (65)	71.3 (57)	
Traditional pit latrine or no facility	14.6 (40)	20.3 (15)	11.7 (14)	13.8 (11)	
Total number of household assets^2^	3.9 ±3.0	3.6 ±3.0	4.6 ±3.2	3.2 ±2.5	0.006
HFIAS score^3^	5.5 ±7.1	5.4 ±7.0	4.1 ±6.2	7.8 ±7.8	0.001
Total amount spent on food for household in a typical week (USD)^4^	$10.61 ±$9.32	$10.78 ±$9.55	$12.22 ±$10.55	$8.05 ±$6.18	0.02
Who purchases most food the family consumes					
Mother	17.2 (47)	16.2 (12)	17.5 (21)	17.5 (14)	0.92
Husband/partner	59.1 (162)	58.1 (43)	58.3 (70)	61.3 (49)	
Both	14.2 (39)	16.2 (12)	15.8 (19)	10.0 (8)	
Other family member	9.5 (26)	9.5 (7)	8.3 (10)	11.3 (9)	
How woman gets to nearest market/shop to purchase food					
Walk	94.5 (256)	93.2 (69)	95.8 (113)	93.7 (74)	0.71
Other^5^	5.5 (15)	6.8 (5)	4.2 (5)	6.3 (5)	
How long it takes to get to nearest market/shop to purchase food (minutes)	37.5 ±41.1	37.8 ±41.7	32.5 ±35.9	44.7 ±46.9	0.77
Purchase special foods for children <5 years in household^6^					
Yes	51.1 (140)	56.8 (42)	41.7 (50)	60.0 (48)	0.02
No	48.9 (134)	43.2 (32)	58.3 (70)	40.0 (32)	
Total amount spent on special foods for children <5 years in household in a typical week (USD)^4^	$8.33±6.12	$7.31±6.21	$9.68±5.65	$7.23±6.35	0.001
Mother’s body size preference for herself					
Underweight silhouette	1.1 (3)	2.7 (2)	0 (0)	1.3 (1)	0.38
Normal weight silhouette	31.4 (86)	23.0 (17)	35.8 (43)	32.5 (26)	
Overweight silhouette	53.7 (147)	58.1 (43)	50.8 (61)	53.8 (43)	
Obese silhouette	13.9 (38)	16.2 (12)	13.3 (16)	12.5 (10)	
Mother’s perception of healthy body size for herself					
Underweight silhouette	0 (0)	0 (0)	0 (0)	0 (0)	0.45
Normal weight silhouette	5.5 (15)	2.7 (2)	8.3 (10)	3.8 (3)	
Overweight silhouette	35.0 (96)	37.8 (28)	34.2 (41)	33.8 (27)	
Obese silhouette	59.5 (163)	59.5 (44)	57.5 (69)	62.5 (50)	
Mother’s body size preference for her child					
Underweight silhouette	2.2 (6)	0 (0)	5.0 (6)	0 (0)	0.03
Normal weight silhouette	32.9 (90)	32.4 (24)	36.7 (44)	27.5 (22)	
Overweight silhouette	39.1 (107)	39.2 (29)	40.0 (48)	37.5 (30)	
Obese silhouette	25.9 (71)	28.4 (21)	18.3 (22)	35.0 (28)	
Mother’s perception of healthy body size for her child					
Underweight silhouette	0.4 (1)	0 (0)	0.8 (1)	0 (0)	0.60
Normal weight silhouette	7.7 (21)	5.4 (4)	10.0 (12)	6.3 (5)	
Overweight silhouette	24.1 (66)	28.4 (21)	24.2 (29)	20.0 (16)	
Obese silhouette	67.9 (186)	66.2 (49)	65.0 (78)	73.8 (59)	
Child diarrhea in last 2 weeks					
Yes	28.2 (77)	23.3 (17)	23.3 (28)	40.0 (32)	0.02
No	71.8 (196)	76.7 (56)	76.7 (92)	60.0 (48)	
Child fever in last 2 weeks					
Yes	40.3 (110)	37.0 (27)	42.5 (51)	40.0 (32)	0.75
No	59.7 (163)	63.0 (46)	57.5 (69)	60.0 (48)	
Child cough in last 2 weeks					
Yes	63.7 (173)	61.6 (45)	62.5 (75)	66.3 (53)	0.81
No	36.6 (100)	38.4 (28)	37.5 (45)	33.8 (27)	
Mother diarrhea in last 2 weeks					
Yes	11.4 (31)	9.6 (7)	10.0 (12)	15.0 (12)	0.47
No	88.6 (242)	90.4 (66)	90.0 (108)	85.0 (68)	
Mother fever in last 2 weeks					
Yes	12.5 (34)	11.0 (8)	11.7 (14)	15.0 (12)	0.71
No	87.6 (239)	89.0 (65)	88.3 (106)	85.0 (68)	
Mother cough in last 2 weeks					
Yes	29.3 (80)	28.8 (21)	29.2 (35)	30.0 (24)	0.99
No	70.7 (193)	71.2 (52)	70.8 (85)	70.0 (56)	

Values are mean ±SD or % (n).

^1^ P-value from chi-square test for categorical variables and Kruskal-Wallis H test (non-parametric alternative to one-way analysis of variance) for continuous variables.

^2^ Including electricity, koloboyi (home-made kerosene lamp), paraffin lamp, radio, television, mattress, sofa set, table and chair(s), refrigerator, watch, bicycle, and mobile telephone.

^3^ Household Food Insecurity Access Scale (HFIAS) ranging from 0 (no food insecurity) to 27 (severe food insecurity).

^4^ Includes food eaten at home and food eaten away from home. Converted to USD using World Bank local currency unit relative to the US dollar for 2018 for Malawi (732.33). Available from: http://wdi.worldbank.org/table/4.16 (accessed 2 October 2019).

^5^ Includes bicycle, motorbike, and minibus.

^6^ Special foods for children are those foods that are bought only for children (<5 years) and are not consumed by other members of the household.

A comparison of demographic and socio-economic characteristics between the dry and rainy season is presented in S2 Table. As compared to the dry season, during the rainy season, household assets were higher (p<0.001) and children were less likely to report a cough in the last 2 weeks (p = 0.01), but food insecurity was higher (p<0.001) and women reported being less likely to purchase special food for children (p<0.01) and more likely to have a fever in the past 2 weeks (p<0.01).

A comparison of dietary intake between mother-child dyads is presented in Table 3 for mothers and Table 4 for children. With respect to dietary intake, mothers and children in dyads with an overweight child had a higher percent of calories from carbohydrate and lower percent of calories from fat compared to dyads with a normal weight child (both p<0.001), which was reflected in the food group data as these mothers also had the highest intake of grains (p<0.001) and their children had the lowest intake of oil/fat (p = 0.01). Children with overweight mothers had higher intake of sweets as compared to children with normal weight mothers (p = 0.08).

**Table 3:** Summary of dietary intake among mothers in Malawi at baseline (dry season) according to mother-child dyad.

	Total (n = 274)	Overweight Mother, Overweight Child (n = 74)	Overweight Mother, Normal Weight Child (n = 120)	Normal Weight Mother, Overweight Child (n = 80)	P-value^1^
Total calories per day	2484 ±1161	2623 ±1297	2340 ±1064	2572 ±1160	0.23
Percent calories from carbohydrate	59.1 ±8.9	61.6 ±8.0	55.9 ±9.0	61.3 ±8.3	<0.001
Percent calories from fat	29.6 ±9.1	26.8 ±8.6	32.6 ±8.8	27.2 ±8.6	<0.001
Percent calories from protein	11.4 ±2.5	11.6 ±2.2	11.1 ±2.6	11.5 ±2.7	0.17
Grains, *g/day*	977 ±449	1074 ±425	839 ±417	1096 ±464	<0.001
Roots/tubers, *g/day*	109 ±141	96 ±109	116 ±142	109 ±164	0.15
Vegetables, *g/day*	316 ±215	303 ±200	317 ±198	326 ±252	0.73
Fruits, *g/day*	79 ±154	111 ±244	58 ±69	81 ±132	0.67
Legumes and nuts, *g/day*	164 ±159	163 ±200	156 ±125	179 ±162	0.72
Meat and eggs, *g/day*	49 ±50	59 ±65	47 ±46	45 ±40	0.66
Fish, *g/day*	45 ±45	47 ±50	42 ±44	47 ±41	0.43
Dairy, *g/day*	21 ± 54	20 ±51	21 ±51	21 ±62	0.69
Oil/fat, *g/day*	17 ±14	15 ±12	18 ±13	17 ±16	0.17
Snacks, *g/day*	54 ±90	46 ±79	58 ±95	55 ±93	0.76
Sweets, *g/day*	135 ±197	151 ±216	130 ±164	130 ±224	0.57

Values are mean ±SD.

^1^ P-value from Kruskal-Wallis H test (non-parametric alternative to one-way analysis of variance).

**Table 4:** Summary of dietary intake among children in Malawi at baseline (dry season) according to mother-child dyad.

	Total (n = 274)	Overweight Mother, Overweight Child (n = 74)	Overweight Mother, Normal Weight Child (n = 120)	Normal Weight Mother, Overweight Child (n = 80)	P-value^1^
Total calories per day	1306 ±886	1327 ±977	1380 ±861	1175 ±831	0.25
Percent calories from carbohydrate	56.8 ±11.3	58.8 ±12.0	54.8 ±11.8	58.0 ±9.3	0.006
Percent calories from fat	33.0 ±11.4	31.0 ±12.2	35.6 ±11.6	31.1 ±9.8	<0.001
Percent calories from protein	10.1 ±3.3	10.2 ±3.1	9.6 ±3.0	10.9 ±3.9	0.05
Grains, *g/day*	326 ±241	352 ±249	307 ±251	330 ±217	0.32
Roots/tubers, *g/day*	37 ±95	35 ±60	41 ±48	34 ±58	0.03
Vegetables, *g/day*	124 ±109	113 ±86	125 ±79	134 ±122	0.38
Fruits, *g/day*	36 ±73	49 ±116	30 ±37	34 ±62	0.31
Legumes and nuts, *g/day*	105 ±109	109 ±123	107 ±101	99 ±110	0.63
Meat and eggs, *g/day*	27 ±36	32 ±53	26 ±29	24 ±27	0.73
Fish, *g/day*	17 ±21	19 ±24	16 ±20	17 ±18	0.52
Dairy, *g/day*	52 ±108	52 ±145	58 ±97	44 ±81	0.76
Oil/fat, *g/day*	14 ±13	12 ±13	17 ±15	11 ±11	0.01
Snacks, *g/day*	53 ±73	52 ±70	60 ±80	45 ±62	0.57
Sweets, *g/day*	98 ±146	109 ±189	113 ±144	67 ±90	0.08

Values are mean ±SD.

^1^ P-value from Kruskal-Wallis H test (non-parametric alternative to one-way analysis of variance).

A comparison of dietary intake between seasons is presented in Table 5 for mothers and Table 6 for children. In terms of differences by season, total caloric intake, the percent of calories from carbohydrates, and intake of grains, vegetables, and fruit was higher for both mothers and children in the rainy season compared to the dry season (all p<0.001). Children also had higher intakes of fish and sweets in the rainy season compared to the dry season (both p<0.001). In contrast, the percent of calories from fat among mothers and children (both p<0.001), intake of roots/tubers among mothers (p<0.01), and intake of dairy among children (p<0.01) was lower in the rainy season compared to the dry season.

**Table 5:** Summary of dietary intake between seasons among mothers in overweight mother-child dyads in Malawi (all dyads combined).

	Dry Season^1^ (n = 240)	Rainy Season (n = 240)	P-value^2^
Total calories per day	2497 ±1144	3148 ±1363	<0.001
Percent calories from carbohydrate	59.5 ±8.4	64.0 ±7.4	<0.001
Percent calories from fat	29.2 ±8.8	24.5 ±7.0	<0.001
Percent calories from protein	11.4 ±2.5	11.6 ±2.1	0.29
Grains, *g/day*	1015 ± 435	1355 ± 569	<0.001
Roots/tubers, *g/day*	103 ± 128	80 ± 118	0.003
Vegetables, *g/day*	317 ± 211	458 ± 287	<0.001
Fruits, *g/day*	78 ± 155	121 ± 173	<0.001
Legumes and nuts, *g/day*	167 ± 164	180 ± 143	0.08
Meat and eggs, *g/day*	49 ± 51	50 ± 50	0.88
Fish, *g/day*	45 ± 44	53 ± 76	0.50
Dairy, *g/day*	20 ± 52	22 ± 56	0.62
Oil/fat, *g/day*	17 ± 13	16 ± 13	0.94
Snacks, *g/day*	53 ± 87	47 ± 79	0.29
Sweets, *g/day*	123 ± 165	136 ± 231	0.89

Values are mean ±SD.

^1^ Restricted to n = 240 dyads with follow-up data from the rainy season to facilitate direct comparison.

^2^ P-value from Wilcoxon signed-rank tests (a non-parametric alternative to a paired t-test).

**Table 6:** Summary of dietary intake between seasons among children in overweight mother-child dyads in Malawi (all dyads combined).

	Dry Season^1^ (n = 240)	Rainy Season (n = 240)	P-value^2^
Total calories per day	1306 ± 870	1745 ± 914	<0.001
Percent calories from carbohydrate	56.7 ± 10.9	61.1 ± 9.0	<0.001
Percent calories from fat	33.0 ± 11.2	28.2 ± 9.3	<0.001
Percent calories from protein	10.3 ± 3.4	10.7 ± 2.5	0.05
Grains, *g/day*	331 ± 238	504 ± 291	<0.001
Roots/tubers, *g/day*	35 ± 52	38 ± 57	0.71
Vegetables, *g/day*	126 ± 95	214 ± 154	<0.001
Fruits, *g/day*	34 ± 73	65 ± 78	<0.001
Legumes and nuts, *g/day*	106 ± 107	118 ± 105	0.11
Meat and eggs, *g/day*	27 ± 38	32 ± 35	0.02
Fish, *g/day*	17 ± 21	26 ± 41	<0.001
Dairy, *g/day*	50 ± 108	34 ± 74	0.008
Oil/fat, *g/day*	14 ± 13	14 ± 13	0.82
Snacks, *g/day*	53 ± 69	63 ± 84	0.07
Sweets, *g/day*	96 ± 144	130 ± 157	<0.001

Values are mean ±SD.

^1^ Restricted to n = 240 dyads with follow-up data from the rainy season to facilitate direct comparison.

^2^ P-value from Wilcoxon signed-rank tests (a non-parametric alternative to a paired t-test.

Results for all model coefficients, 95% confidence intervals, and p-values from multivariate logistic regression models of dietary outcomes (caloric, macronutrient, and food group intake) are provided in S1 File. Significant drivers of caloric and macronutrient intake are presented in Table 7. In the rainy season, distance to the nearest market was associated with lower caloric intake for mothers, lower household food insecurity was associated with higher caloric intake among mothers and children, and higher household spending on special foods for children was associated with higher caloric intake among mothers and children. Preference for grains and sweets was positively associated with caloric intake among mothers in the dry season, and preference for fruits was positively associated with caloric intake among mothers in the rainy season. Age was positively associated with caloric intake among children in both the dry and rainy season.

**Table 7:** Significant (p<0.05) predictors of caloric and macronutrient intake during the dry and rainy seasons in Malawi, from multivariate models adjusted for all predictors significant (p<0.10) in univariate models.

Driver of food consumption	Total calories				Percent calories from carbohydrate				Percent calories from fat				Percent calories from protein			
	Mother		Child		Mother		Child		Mother		Child		Mother		Child	
	Dry	Rainy	Dry	Rainy	Dry	Rainy	Dry	Rainy	Dry	Rainy	Dry	Rainy	Dry	Rainy	Dry	Rainy
***Mother-child dyad***																
Overweight mother, normal weight child (vs. both overweight)																
***Accessibility***																
Rural (vs. urban)																
Minutes to nearest food market/shop																
***Affordability***																
Drinking water from well (vs. piped)																
Ventilated pit latrine (vs. flush toilet)																
Pit latrine with roof (vs. flush toilet)																
Traditional pit latrine (vs. flush toilet)																
Number of household assets																
HFIAS score																
Amount spent on special foods for children																
***Desirability***																
Taste preference: grains																
Taste preference: legumes																
Taste preference: fruit																
Taste preference: dairy																
Taste preference: sweets																
Purchase special foods for children																
***Other factors***																
Age of child																
Female sex of child (vs. male)																
Household size																
Mother cough in last 2 weeks																

Black is inversely associated, grey is positively associated.

The drivers of intake of healthy plant-based food groups (grains, roots/tubers, and legumes and nuts) are presented in Table 8. Women with a normal weight child consumed less grains than women with an overweight child during both seasons. Taste preference was consistently positively associated with intake of grains in both mothers and children across seasons, and older child age was positively associated with higher intake of this food group among children. Women who had to spend more time traveling to the nearest food market/shop and women who preferred a body size of normal weight, overweight, or obese (versus underweight) were less likely to consume grains in the rainy season. There were few significant predictors of root/tuber or legume/nut intake. More household spending on special foods for children and older child age were associated with higher intake of roots/tubers among children in the dry and rainy seasons, respectively. Only taste preference for legumes was positively associated with intake in both mothers and children consistently across seasons. Lower household food insecurity was associated with higher legume/nut intake, but only among mothers in the rainy season.

**Table 8:** Significant (p<0.05) predictors of healthy plant-based food groups (grams) during the dry and rainy seasons in Malawi, from multivariate models adjusted for all predictors significant (p<0.10) in univariate models.

Driver of food consumption	Grains				Roots/ tubers				Legumes/nuts			
	Mother		Child		Mother		Child		Mother		Child	
	Dry	Rainy	Dry	Rainy	Dry	Rainy	Dry	Rainy	Dry	Rainy	Dry	Rainy
***Mother-child dyad***												
Overweight mother, normal weight child (vs. both overweight)												
***Accessibility***												
Minutes to nearest food market/shop												
***Affordability***												
HFIAS score												
***Desirability***												
Taste preference: grains												
Taste preference: legumes												
Purchase special foods for children												
Preferred body size of mother: normal weight (vs. underweight)												
Preferred body size of mother: overweight (vs. underweight)												
Preferred body size of mother: obese (vs. underweight)												
Perceived healthy body size of child: overweight (vs. underweight)												
Perceived healthy body size of child: obese (vs. underweight)												
***Other factors***												
Age of child												
Mother diarrhea in last 2 weeks												

Black is inversely associated, grey is positively associated.

The drivers of intake of fruits and vegetables are presented in Table 9. Taste preference for vegetables predicted higher vegetable intake among mothers and children in both seasons. Older children also had higher vegetable intake in both seasons. In the rainy season, for both mothers and children, a normal weight child predicted lower vegetable intake while having a borehole well predicted higher vegetable intake. Mothers with lower autonomy who primarily bought food for the house (vs. other family member) also reported lower vegetable intake for their children in the rainy season. In the dry season, lower spending on special foods for children predicted higher vegetable intake. There were few significant predictors of fruit intake. The only predictor of child fruit intake was household assets: higher assets predicted higher fruit intake among children in the rainy season. Among mothers in the rainy season, having a normal weight child, living in Lilongwe (vs. Kasungu), and having piped water (vs. borehole well) predicted higher fruit intake, while in the dry season, less food insecurity predicted higher fruit intake.

**Table 9:** Significant (p<0.05) predictors of vegetables and fruits (grams) during the dry and rainy seasons in Malawi, from multivariate models adjusted for all predictors significant (p<0.10) in univariate models.

Driver of food consumption	Vegetables				Fruits			
	Mother		Child		Mother		Child	
	Dry	Rainy	Dry	Rainy	Dry	Rainy	Dry	Rainy
***Mother-child dyad***								
Overweight mother, normal weight child (vs. both overweight)								
***Accessibility***								
Kasungu district (vs. Lilongwe)								
Other family member primarily buys food (vs. mother)								
Female autonomy								
***Affordability***								
Well borehole (vs. piped)								
Number of household assets								
HFIAS score								
Amount spent on special foods for children								
***Desirability***								
Taste preference: vegetables								
***Other factors***								
Age of child								

Black is inversely associated, grey is positively associated.

The drivers of intake of animal-source foods are presented in Table 10. There were many predictors of meat and eggs as well as dairy, but few for fish intake. For meat and eggs, during the dry season, more household assets predicted higher intake among both children and mothers and older child age predicted higher intake among children. In addition, mothers who purchased the majority of food for the household and who used mechanized transport instead of walking to buy food had higher intakes of meat and eggs. During the rainy season, households that spent more on food including special foods for children, those with lower food insecurity, and those with female children consumed more meat and eggs. Living in a rural (vs. urban) area predicted lower fish intake and maternal taste preference and older child age predicted higher fish intake among children during the dry season. Shorter travel time to the nearest market/shop predicted higher fish intake among mothers in the rainy season. The amount spent on food predicted higher dairy intake for mothers and children in the dry and rainy season, respectively. Similarly, purchasing special foods for children and the amount spent on special foods for children predicted higher dairy intake in mothers during the dry season. The amount spent on special foods for children predicted higher dairy intakes among children in the rainy season. Mothers who thought the underweight child silhouette was healthier than the overweight or obese silhouette had higher intakes of dairy during the dry season, as did their children.

**Table 10:** Significant (p<0.05) predictors of animal-based food groups (grams) during the dry and rainy seasons in Malawi, from multivariate models adjusted for all predictors significant (p<0.10) in univariate models.

Driver of food consumption	Meat and eggs				Fish				Dairy			
	Mother		Child		Mother		Child		Mother		Child	
	Dry	Rainy	Dry	Rainy	Dry	Rainy	Dry	Rainy	Dry	Rainy	Dry	Rainy
***Accessibility***												
Rural (vs. urban)												
Minutes to nearest food market/shop												
Other family member primarily buys food (vs. mother)												
Other transport to buy food (vs. walk)												
***Affordability***												
Number of household assets												
HFIAS score												
Amount spent on food for household												
Amount spent on special foods for children												
***Desirability***												
Taste preference: fish												
Purchase special foods for children												
Perceived healthy body size of mother: obese (vs. underweight)												
Perceived healthy body size of child: normal weight (vs. underweight)												
Perceived healthy body size of child: overweight (vs. underweight)												
Perceived healthy body size of child: obese (vs. underweight)												
***Other factors***												
Age of child												
Female sex of child (vs. male)												
Child fever in last 2 weeks												
Mother cough in last 2 weeks												

Black is inversely associated, grey is positively associated.

Table 11 presents significant predictors of oil/fat, snacks, and sweets, with sweets having the greatest number of predictors. Higher household assets predicted higher intakes of oil/fat among mothers and children during the dry season, and lower food insecurity predicted higher intake of high oil/fat among mothers and children during the rainy season. For mothers during the rainy season, less travel time to a shop predicted higher oil/fat consumption. Children whose mother and father both buy food (versus just the mother) and older children had higher oil/fat intakes during the dry season. Children whose mother did not have diarrhea in the past two weeks and maternal taste preference for oil/fat positively predicted child intake of this food group during the rainy season. Greater spending on special foods and lower household food insecurity predicted higher snack intake in both mothers and children across seasons. Older children and those living in urban areas consumed more snacks in the dry and rainy seasons, respectively. Mothers who used some form of mechanized transport instead of walking to purchase food and those with more household assets had higher intakes of sweets in the dry and rainy seasons, respectively. During the dry season, children whose homes had a flush toilet, who had special foods purchased for them, whose family’s spent more money on special foods, and whose mother’s perceived overweight or obese silhouettes to be healthier, who were older, and whose mothers had secondary education or higher had higher intakes of sweets. Similar predictors of child sweet intake were observed in the rainy reason, except that the opposite association was observed for mother’s perception of a healthy body size for the child.

**Table 11:** Significant (p<0.05) predictors of unhealthy food groups (grams) during the dry and rainy seasons in Malawi, from multivariate models adjusted for all predictors significant (p<0.10) in univariate models.

Driver of food consumption	Oil/fat				Snacks				Sweets			
	Mother		Child		Mother		Child		Mother		Child	
	Dry	Rainy	Dry	Rainy	Dry	Rainy	Dry	Rainy	Dry	Rainy	Dry	Rainy
***Mother-child dyad***												
Overweight mother, normal weight child (vs. both overweight)												
Normal weight mother, overweight child (vs. both overweight)												
***Accessibility***												
Rural (vs. urban)												
Minutes to nearest food market/shop												
Both parents primarily buy food (vs. mother)												
Other transport to buy food (vs. walk)												
***Affordability***												
Ventilated pit latrine (vs. flush toilet)												
Pit latrine with roof (vs. flush toilet)												
Traditional pit latrine (vs. flush toilet)												
Number of household assets												
HFIAS score												
Amount spent on special foods for children												
***Desirability***												
Taste preference: oil/fat												
Purchase special foods for children												
Perceived healthy body size of mother: overweight (vs. underweight)												
Preferred body size of mother: obese (vs. underweight)												
Perceived healthy body size of child: normal weight (vs. underweight)												
Perceived healthy body size of child: overweight (vs. underweight)												
Perceived healthy body size of child: obese (vs. underweight)												
***Other factors***												
Age of child												
Mother secondary education or higher (vs. less)												
Mother diarrhea in last 2 weeks												

Black is inversely associated, grey is positively associated.

## Discussion

In this study, drivers of food consumption varied between mothers and children, and across seasons and food groups. Food affordability and desirability as well as other factors, particularly child age, were the most consistent predictors of food consumption. Within the domain of affordability, household food insecurity was associated with lower intake of grains, fruits, meat and eggs, oil/fat, and snacks, and household food expenditures were associated with higher animal-source food consumption. Within the domain of desirability, maternal taste preferences predicted increased consumption of grains, legumes/nuts, vegetables, fish, and oil/fat, and maternal body size preferences for herself and her child were associated with consumption of grains, legumes/nuts, dairy, and sweets. Food accessibility was also a predictor for some food groups. Having to spend less time traveling to the market/shops, using some form of mechanized transport instead of walking to the market/shops, and the mother purchasing foods (versus some other family member) tended to be positively associated with intake of foods such as grains, vegetables, meat and eggs, oil/fat, and sweets.

These findings align with previous research among women in Africa showing that elements of food affordability, including socioeconomic status and household food expenditures, and food desirability, including taste and body size perceptions, are related to different aspects of dietary behavior [21]. This study extends the previous research by examining associations of predictors by season and for children. Previous studies have found that women in Malawi and other parts of SSA living in urban areas and those who have higher socioeconomic status are more likely to be overweight [13, 21, 33–35]. Perhaps because our study specifically enrolled mother-child dyads with an overweight member across urban and rural areas, urban/rural residence was associated with few dietary outcomes or food groups. Most of the participants in our study had low socioeconomic status based on their number of household assets, which explains why food affordability, measured through food insecurity, food expenditures, and socioeconomic indicators, was associated with food consumption.

Previous studies have linked seasonal variation in food availability to dietary intake in rural areas [6]. In this study, we observed that total caloric intake, the percent of calories from carbohydrates, and intake of grains, vegetables, and fruit were higher in the rainy season compared to the dry season, and percent of calories from fat and intake of roots/tubers were lower. Maize, the staple, is consumed daily by most Malawians and is the biggest component of caloric intake and the most likely driver of seasonal variations in weight of young children [36]. Maize is plentiful in the harvest period (April-May) toward the end of the rainy season and stocks of staples and other food dwindle early in the rainy season (November-February). Maize is harvested in April and May, but people start eating it when its fresh, that is, from March. Most of our rainy season data were collected in March and April, which explains why intake of grains and calories from carbohydrates was higher in the rainy than dry season in this study. Fruit and vegetable production in Malawi is dependent on rain-fed agriculture; therefore, fruits and vegetables are more readily available, less expensive, and more frequently consumed during the middle and later parts of the rainy season.

Our systematic review of the literature (S1 Text, S1 Fig, and S3 Table) found that most previous studies on drivers of food consumption in SSA focused on fruit and vegetable intake [21, 37–55]. We did not find many significant predictors of fruit intake in our study, but in the rainy season, food affordability, particularly indicators of higher wealth, predicted higher fruit intake in both mothers and children. Living in the capital city Lilongwe predicted higher fruit intake in mothers. Economically, people in Lilongwe are better off and are more able to purchase fruits as compared to those in Kasungu. This is largely consistent with what previous studies have found: seven of the nine studies on fruit intake reported a positive association with wealth [44, 45, 47–49, 51, 52]. The reason for this may be that fruits are expensive and perishable [53, 56, 57], and in Malawi, households purchase rather than produce the majority of the fruits they consume.

In contrast, for vegetables, indicators of food affordability were not strong predictors in our study, and instead, mothers who primarily purchased food for the household (versus another family member) and spent less on special foods for children had higher vegetable intakes. Green leafy vegetables (which are the most commonly consumed vegetable) are inexpensive and most households can afford them. This trend for vegetables is consistent with the literature found in our review: findings on assets as a predictor of vegetable intake were inconclusive. Three of five studies reported that higher income predicted higher vegetable intake [44, 47, 48] while the remaining two found that low assets predicted higher intake [37, 38].

With respect to animal-source foods, we found that mother-child dyads with more assets and who spent more on food, indicators of food affordability, had higher intakes of meat, eggs, and dairy. Our literature review found that four of six studies on income found that higher assets/income predicted higher meat consumption [21, 39, 45–48]. We found that two of four studies on consumption by area of residence predicted higher intake of meat among those living in urban areas [43, 45, 47, 48]. However, urban residence was not associated with meat intake in our study but was associated with higher fish intake among children. There is a general consensus that as economic development and urbanization have progressed, intake of animal-source foods increases [58]. This likely relates to the fact that animal-source foods are expensive, and only become affordable as purchasing power increases.

With regards to unhealthy food groups such as snacks and sweets, we found that higher food insecurity, an indicator of food affordability, predicted lower intake of these foods. The literature on unhealthy foods from our review suggested that living an urban lifestyle (full-time job but low wages, piped water, electricity, taste preference for fast food) predicted high intake of unhealthy, processed foods [21, 53, 54]. Our findings that unhealthy body size preferences, an indicator of desirability, predicted higher snack and sweet intake are also supported by literature from our review, which suggest that body image and lack of dietary knowledge contribute to higher levels of consumption of unhealthy foods [21, 53, 54]. Importantly, we also observed that dyads with overweight children were more likely to purchase special foods for children and spend more money on these special foods. Previous studies found that Malawian parents in rural areas have a preference for packaged foods, like biscuits, Kamba puffs (a packaged snack food), and orange soda, over less processed, more nutrient dense alternatives [59–62]. Together with our findings, this suggests that behavior change communication interventions targeting feeding of “junk” foods to young children could be important for tackling the obesity epidemic in this setting.

This is the first study to empirically measure how taste preferences, an important indicator of desirability, predict food consumption in SSA. Even after controlling for wealth, area of residence, and other factors, taste preferences predicted high consumption of grains, legumes, vegetables, fish, and oil/fat. Literature found in our systematic review was consistent with this finding, although all studies that identified taste as a driver of food consumption were qualitative [39, 40, 42, 43, 46, 54]. The one systematic review located in our literature review found only one study on taste published in 2004 [21]. Work previously conducted in Malawi studied favorite tastes of foods and supplements among those living with HIV, but research on interventions regarding taste is lacking [63]. Given the consistent associations between taste and dietary intake in our study, more research into how taste preferences are formed and how to modify taste preferences from an early age in order to promote healthier diets and prevent obesity in the SSA context is needed.

## Conclusions

This study confirmed that previously explored drivers such as household expenditures on food, maternal body size preferences, and child age are important drivers of dietary intake. We also report novel findings regarding the importance of maternal taste preferences, spending on “special” foods for children, time spent traveling to the market/shops, using some form of mechanized transport instead of walking to the market/shops, and who purchases the food (the mother versus some other family member). Our findings that household expenditures and taste preferences are predictors of food consumption align with our qualitative results from this study, which showed that cost and taste are key drivers in this population [59]. Given that overweight in Malawi is contributing to increasing prevalence of diabetes and increasing risk of death from cardiac diseases [60, 61], these results can be used for designing food policies and planning interventions to prevent or address overweight in Malawian mothers and children. Future research should investigate the efficacy of interventions that target specific drivers identified in this study to prevent overweight in mothers and children in Malawi.

## Supporting information

S1 Fig Selection process for a systematic review of studies regarding drivers of food choice in Sub-Saharan Africa. (PNG)

S1 Table Summary of food items contributing to 11 food groups included in analysis. (DOCX)

S2 Table Summary of demographic and socio-economic characteristics between seasons among overweight mother-child dyads in Malawi (all dyads combined). (DOCX)

S3 Table Relevant studies identified in review of literature of PubMed and Google Scholar on drivers of food choice in sub-Saharan Africa. (DOCX)

S1 Text Methodology for systematic review. (DOCX)

S1 File Multivariate linear regression model estimates. (DOCX)
