# Supplementary figures and images for: Drivers of food consumption among overweight mother-child dyads in Malawi
Source: PLoS One. 2020 Dec 17;15(12):e0243721. doi: 10.1371/journal.pone.0243721 (PMC7745992; doi:10.1371/journal.pone.0243721)

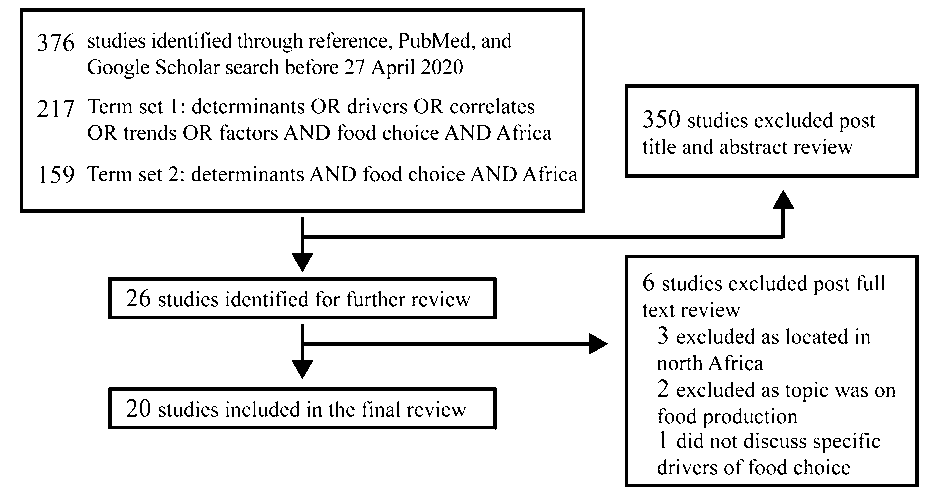

Supplement: S1 Fig — (PNG) [file pone.0243721.s001.png]
