# Supplementary material for: Drivers of food consumption among overweight mother-child dyads in Malawi
Source: PLoS One. 2020 Dec 17;15(12):e0243721. doi: 10.1371/journal.pone.0243721 (PMC7745992; doi:10.1371/journal.pone.0243721)
Supplement: S1 Table — (DOCX) [file pone.0243721.s002.docx]

| **S1 Table.** Summary of food items contributing to 11 food groups included in analysis. | |
| --- | --- |
| Food group | Items from quantitative food frequency questionnaire included |
| Grains | White bread, buns/scones  African bread  Doughnut, fritter/mandazi, wheat dough deep fried  Green maize  Nsima, mgaiwa, stiff maize porridge made from whole grain flour  Nsima, ufawoyera, stiff maize porridge made from processed flour  Nsima, gramil, stiff maize porridge made from partially processed flour  Nsima, madeya, stiff porridge made from flour from maize husks  Rice  Phala, mgaiwa, maize porridge made from whole grain flour  Phala, ufawoyera, maize porridge made from processed flour  Rice phala, porridge made from rice  Likuni phala, porridge made from micronutrient-fortified corn soy flour blend  Cerelac, nestum, or other commercially prepared porridge  Spaghetti/pasta/macaroni  Breakfast cereals, like cornflakes or other packaged cereals  Maize grits |
| Roots/tubers | Irish potato  Sweet potato  Cassava  Cocoyam  Plantain |
| Vegetables | Cabbage salad  Boiled cabbage  Cucumber  Eggplant, stewed in relish  Green/red pepper, stewed in relish  Green beans, stewed in relish  Carrot, raw  Carrot, stewed in relish  Tomato, stewed in relish  Mushroom, stewed in relish  Pumpkin, boiled  Green leafy vegetable relish, plain  Green leafy vegetable relish with groundnut powder  Green leafy vegetable relish with oil  Green leafy vegetable relish with groundnut powder and oil  Okra relish  Onions  Hibiscus  Gourd |
| Fruits | Avocado  Banana  Baobab  Tamarind  Coconut  Custard apple  Guava  Lemon, lime, orange, tangerine  Mango  Masau  Loquat  Pawpaw  Passion fruit, granadilla  Pineapple  Watermelon  Flacourtina Indica |
| Meat/eggs | Roasted chicken, chicken liver, chicken gizzard, dove, pigeon, guinea fowl, duck  Fried chicken, chicken liver, chicken gizzard, dove, pigeon, guinea fowl, duck  Stewed chicken, chicken liver, chicken gizzard, dove, pigeon, guinea fowl, duck  Roasted pork  Fried pork  Stewed pork in relish  Roasted goat, goat offals  Fried goat, goat offals  Stewed goat in relish  Roasted sheep  Fried sheep  Stewed sheep in relish  Sausage  Roasted mice, rats  Roasted guinea pig  Egg |
| Fish | Roasted medium or large fresh fish  Fried medium or large fresh fish  Stewed medium or large fresh fish  Fried medium or large dried fish  Stewed medium or large dried fish  Roasted small fresh fish  Fried small fresh fish  Stewed small fresh fish  Fried small dried fish  Stewed small dried fish |
| Dairy | Yoggie (liquid yoghurt), yoghurt  Cheese  Fresh milk  Milk powder |
| Legumes and nuts | Fresh pea/bean relish, plain  Fresh pea/bean relish with groundnut flour  Fresh pea/bean relish with oil  Fresh pea/bean relish with groundnut flour and oil  Dried pea/bean relish, plain  Dried pea/bean relish with groundnut flour  Dried pea/bean relish with oil  Dried pea/bean relish with groundnut butter and oil  Soup made with groundnuts  Fresh, boiled groundnuts  Dried, roasted groundnuts  Raw groundnuts  Peanut butter  Sibusiso, fortified ready-to-use food made of groundnuts  Soya pieces  Soya phala, porridge made from soy flour  Cashew nut  Pumpkin seeds, sunflower seeds |
| Oil/fat | Butter, margarine  Cooking oil |
| Snacks | Chips, french fries  Kamba puffs, packaged maize snakcs  Potato chips, crisps  Samosa  Maize popcorn  Jigs/jiggies, packaged maize snacks  Zigege, Irish potato fritters |
| Sweets | Biscuits  Custard  Freezes, popsicles  Jam  Brown sugar, white sugar  Sugarcane  Candies/sweets/lollipop  Chocolate  Honey  African cake  Other cakes and pastries  Ice cream  Fruit juice  Fizzy drinks, soda, including Sobo, Frozy, Coke, Fanta, Sprite |
