# Supplementary material for: Drivers of food consumption among overweight mother-child dyads in Malawi
Source: PLoS One. 2020 Dec 17;15(12):e0243721. doi: 10.1371/journal.pone.0243721 (PMC7745992; doi:10.1371/journal.pone.0243721)
Supplement: S2 Table — (DOCX) [file pone.0243721.s003.docx]

| **S2 Table**. Summary of demographic and socio-economic characteristics between seasons among overweight mother-child dyads in Malawi (all dyads combined). | | | |
| --- | --- | --- | --- |
|  | Dry Season^1^  (n=240) | Rainy Season  (n=240) | P-value^2^ |
| Main source of drinking water |  |  |  |
| Piped into dwelling, plot/yard, or communal standpipe | 55.8 (134) | 54.4 (130) | 0.24 |
| Well in yard/plot or public well | 12.1 (29) | 9.2 (22) |  |
| Borehole | 32.1 (77) | 36.4 (87) |  |
| Toilet facility used by household |  |  |  |
| Flush toilet | 5.4 (13) | 2.9 (7) | 0.06 |
| Ventilated improved pit latrine | 16.7 (40) | 20.8 (50) |  |
| Pit latrine with roof | 62.9 (151) | 66.3 (159) |  |
| Traditional pit latrine or no facility | 15.0 (36) | 10.0 (24) |  |
| Total number of household assets^3^ | 3.8 ± 3.0 | 4.3 ± 3.0 | <0.001 |
| HFIAS score^4^ | 5.7 ± 7.2 | 7.8 ± 7.4 | <0.001 |
| Total amount spent on food for household in a typical week (USD)^5^ | $10.28 ± 9.33 | $9.96 ± 8.66 | 0.72 |
| Who purchases most food |  |  |  |
| Mother | 17.5 (42) | 15.8 (38) | 0.46 |
| Husband/partner | 60.4 (145) | 59.6 (140) |  |
| Both | 12.9 (31) | 15.8 (37) |  |
| Other family member | 9.2 (22) | 8.8 (21) |  |
| How woman gets to nearest market/shop to purchase food |  |  |  |
| Walk | 95.4 (226) | 94.5 (225) | 0.82 |
| Other^6^ | 4.6 (11) | 5.5 (13) |  |
| How long it takes to get to nearest market/shop to purchase food (minutes) | 39.1 ± 42.4 | 23.2 ± 33.0 | <0.001 |
| Purchase special foods for children <5 years in household^7^ |  |  |  |
| Yes | 52.1 (125) | 36.4 (86) | <0.01 |
| No | 47.9 (115) | 63.6 (150) |  |
| Total amount spent on special foods for children <5 years in household in a typical week (USD) | $8.24 ± 6.16 | $1.38 ± 2.12 | <0.0001 |
| Child diarrhea in last 2 weeks |  |  |  |
| Yes | 27.6 (66) | 28.3 (68) | 0.91 |
| No | 72.4 (173) | 71.7 (172) |  |
| Child fever in last 2 weeks |  |  |  |
| Yes | 41.0 (98) | 45.4 (109) | 0.32 |
| No | 59.0 (141) | 54.6 (131) |  |
| Child cough in last 2 weeks |  |  |  |
| Yes | 64.4 (154) | 52.7 (126) | 0.01 |
| No | 35.6 (85) | 47.3 (113) |  |
| Mother diarrhea in last 2 weeks |  |  |  |
| Yes | 10.5 (25) | 9.6 (23) | 0.10 |
| No | 89.5 (214) | 90.4 (217) |  |
| Mother fever in last 2 weeks |  |  |  |
| Yes | 13.4 (32) | 22.9 (55) | <0.01 |
| No | 86.6 (207) | 77.1 (185) |  |
| Mother cough in last 2 weeks |  |  |  |
| Yes | 29.3 (70) | 28.0 (67) | 0.74 |
| No | 70.7 (169) | 72.0 (172) |  |
| Values are mean ±SD or % (n).  ^1^ Restricted to n=240 dyads with follow-up data from the rainy season to facilitate direct comparison.  ^2^ P-value from exact test for symmetry for categorical variables and Wilcoxon signed-rank tests (a non-parametric alternative to a paired t-test) for continuous variables.  ^3^ Including electricity, koloboyi (home-made lamp), paraffin lamp, radio, television, mattress, sofa set, table and chair(s), refrigerator, watch, bicycle, and mobile telephone.  ^4^ Household Food Insecurity Access Scale (HFIAS) ranging from 0 (no food insecurity) to 27 (severe food insecurity).  ^5^ Includes food eaten at home and food eaten away from home. Converted to USD using World Bank local currency unit relative to the US dollar for 2018 for Malawi (732.33). Available from: http://wdi.worldbank.org/table/4.16 (accessed 2 October 2019).  ^6^ Includes bicycle, motorbike, and minibus.  ^7^ Special foods for children are those foods that are bought only for children (<5 years) and are not consumed by other members of the household. | | | |
