# Supplementary material for: Drivers of food consumption among overweight mother-child dyads in Malawi
Source: PLoS One. 2020 Dec 17;15(12):e0243721. doi: 10.1371/journal.pone.0243721 (PMC7745992; doi:10.1371/journal.pone.0243721)
Supplement: S3 Table — (DOCX) [file pone.0243721.s004.docx]

| **S3 Table.** Relevant studies identified in review of literature of PubMed and Google Scholar on drivers of food choice in sub-Saharan Africa. | | | | | | | | | |
| --- | --- | --- | --- | --- | --- | --- | --- | --- | --- |
| First Author | Year | Demographic | Food groups | Study type | n Males | n Females | Tests | Predictors of increased intake |  |
| Joshua Amo-Adjei | 2014 | adults in Ghana | fruits, vegetables | cross sectional survey | 4916 | 4568 | Poisson regression | Living in forested region (FV)*, lower assets (V)*, education (F)* |  |
| Fusta Azupogo | 2018 | women in Ghana | vegetables | cross sectional survey | 0 | 187 | generalized linear models | Low assets, high quality of life, high physical, emotional, and mental health |  |
| Corbett Brown | 2015 | adolescents in Botswana | all food | qualitative focus groups | 72 | 72 | none | Taste, social stigma, and low assets cause parents to feed their kids unhealthy foods |  |
| Gamuchirai Chakona | 2019 | pregnant women in South Africa | all food | qualitative focus groups | 0 | 224 | none | Certain vitamin A and mineral rich foods are eschewed during pregnancy due to culture preferences and taboos |  |
| Alisha Farris | 2019 | Malagasy people | all food | qualitative focus groups | 0 | 137 | None | Cleanliness, taste, price, perceived healthfulness, availability/seasonality |  |
| Stefanie C Gissing | 2016 | woman in urban areas in SSA countries | all food, focus on predictors of consumption of unhealthy foods | systematic review | 0 | 4347 | none | Negative body image, poor food knowledge, skipping meals, high alcohol consumption, old age, high assets, high education, low food expenditure |  |
| Anita Heim | 2019 | San people in Namibia | grains, beans, seeds, dairy, meat, eggs, leafy V, vitamin A FV | cross sectional survey | 89 | 111 | Spearman, Mann Whitney U | Most participants only consumed foods in 2/3 of the food groups. Age predicted higher diet diversity score |  |
| Anita Heim | 2020 | San people in Namibia | all food | qualitative focus groups | 69 | 89 | none | Taste for food, high health, low hunger, high food access, and seasonality predicted high healthy food consumption |  |
| Jo Hunter-Adams | 2019 | people adapting to urban environment in South Africa | all food | qualitative focus groups | 1 | 77 | none | Low food security, living in an urban area, lack of access, and social stigma predicted unhealthy food intake. Low consumption of vegetables despite preference |  |
| Beverly Msambichaka | 2018 | ≥15 years in Tanzania | fruits, vegetables | cross sectional survey | 2839 | 5114 | chi squared, odds ratios | High education (F), old age, being female, high education, high income, low tobacco, and high primary care (FV) |  |
| Andrew Muhammad | 2017 | 2010 Global Dietary Database on 45 countries in SSA | fruits, vegetables, beans, legumes, nuts, seeds, whole grains, processed and unprocessed meat, fish, dairy, high sugar drinks | cross sectional survey | n/a | n/a | semi-log quadratic functional model | High income predicted high processed meat intake in younger men and women. High income predicted high fruit consumption in all demographics |  |
| Eunice Nago | 2012 | adolescents in urban areas in Benin | all food | qualitative focus groups | 70 | 83 | none | High availability, accessibility, nutritional education, taste, and low availability of unhealthy foods (FV) |  |
| Kufre Joseph Okop | 2019 | men and women in low-income areas in South Africa | cereal, starch, meat, vegetables, fruit, bread, sugary drinks, snacks, sugar | cross sectional survey | 124 | 411 | Pearson Chi square tests, ANOVA, multivariate logistic regression models | Urban environment (F, meat), purchasing sweet drinks, high income, and owning a car (FV). Education and taste did not predict FV |  |
| Chioma Sylvia Okoro | 2017 | construction workers in South Africa | Dairy, eggs, nuts, fish, cereal, salty food, sugary food, fried food, pasta, grains like rice, fruits, vegetables, meat, corn meal | cross sectional survey | 163 | 20 | Principal components and multiple regression analysis | High income, assets, and coming from rural areas (FV), nutritional knowledge and high assets (dairy, meat) |  |
| Karl Peltzer | 2012 | older adults in South Africa | fruits, vegetables | cross sectional survey | 1693 | 2147 | post stratified individual probability, odds ratios | Being part of the white population group, high education, and no tobacco use (FV) |  |
| Karl Peltzer | 2015 | university students in SSA countries | fruits, vegetables | cross sectional survey | 400 | 400 | univariate and multivariate logistic regression | High self-esteem, body image, assets, taste for FV, low alcohol, high physical activity increased FV |  |
| Supa Pengpid | 2015 | adults in Kenya | fruits, vegetables | cross sectional survey | 1792 | 2687 | odds ratios | Living in rural area and being from the Kikuyu and Lo peoples (FV), being above age 29 (V), assets and primary school or higher education (F) |  |
| Marie Ruel | 2005 | adults in 10 SSA countries | fruits, vegetables | analysis of data from multiple surveys | n/a | n/a | regression models | Urban areas in wealthy countries (Kenya) (FV), other countries no significant difference in FV for urban/rural |  |
| Nelia Steyn | 2011 | ≥16 years in South Africa | street food, fast food | cross sectional survey | 1338 | 1952 | logistic regression | Blacks purchased most street food, fruit was most common street food. Owning home appliances, full time job, living in a house, low wages, lack of pension, having piped water and electricity (fast food) |  |
| Carlijn Voorend | 2012 | female adolescents in South Africa | all food, focus on fast/street food | qualitative focus groups | 0 | 58 | none | Eating at school, perception of high cost, low quality, and preference predicted unhealthy food intake |  |

* Fruits and vegetables abbreviated as FV, fruits as F, vegetables as V.
