# Supplementary material for: Drivers of food consumption among overweight mother-child dyads in Malawi
Source: PLoS One. 2020 Dec 17;15(12):e0243721. doi: 10.1371/journal.pone.0243721 (PMC7745992; doi:10.1371/journal.pone.0243721)
Supplement: S1 Text — (DOCX) [file pone.0243721.s005.docx]

**S1 Text. Methodology for systematic review.**

We conducted a systematic literature review of papers published from 2010 to present on drives of food choice in sub-Saharan Africa (SSA) using PubMed and Google Scholar (**S1 Fig**). Eligibility criteria were as follows conducted in sub-Saharan Africa, used either quantitative surveys or qualitative interviews or focus groups, analyzed factors affecting patterns of consumption, and included at least one of the food groups of interest (fruits, vegetables, meat, dairy, grains, legumes, and “junk” foods including sugar-sweetened beverages). Meta-analyses were included as a single study if none of its included studies appeared in our search. Using MeSH terms, two groups of keywords were used: 1) determinants OR drivers OR correlates OR trends OR factors AND food choice AND Africa 2) determinants AND food choice AND Africa. After running the same keywords translated in Arabic, French, and Spanish using Google Translate, 217 and 159 potentially relevant studies, including one Spanish language study, were identified for the first and second group of keywords, respectively. Twenty-five studies were included after title and abstract review. After full text review, three were excluded as they took place in North Africa, one was excluded as it did not discuss specific drivers of food choice, and two were excluded as they were reviews of food production. A total of 20 papers were thus included in this review (**S3 Table**).

Ten of the studies specifically surveyed vegetable consumption, nine surveyed fruit consumption, six surveyed meat consumption, and three surveyed fast food consumption. Seven of the nine studies on fruit found that high income and assets predicted high fruit intake. Patterns of fruit consumption by urban and rural area were less conclusive, with three studies predicting higher intake in rural areas while one predicted higher intake in urban. In studies surveying vegetables, high income, low assets, and living in rural areas predicted high intake in three, two, and four studies, respectively. High assets predicted high meat consumption in four of six studies, while living in an urban area predicted high meat intake in two. All studies on fast food found that there was a social stigma against traditional meals and higher social status associated with processed food. Low wages also predicted high fast food intake.
