# Supplementary material for: Drivers of food consumption among overweight mother-child dyads in Malawi
Source: PLoS One. 2020 Dec 17;15(12):e0243721. doi: 10.1371/journal.pone.0243721 (PMC7745992; doi:10.1371/journal.pone.0243721)
Supplement: S1 File — (DOCX) [file pone.0243721.s006.docx]

| **Table 1.** Predictors of caloric intake among mothers in the dry season. Model estimates from multivariate linear regression. P<0.05 are bold. | | | | |
| --- | --- | --- | --- | --- |
| **Variable** | **Coefficient** | **P-value** | **Lower 95% CI** | **Upper 95% CI** |
| Rural (vs. urban) | -67.84 | 0.75 | -484.73 | 349.04 |
| Mother secondary education or higher (vs. less) | 135.36 | 0.35 | -151.96 | 422.69 |
| Main source of drinking water |  |  |  |  |
| Well (vs. piped) | -408.57 | 0.09 | -881.04 | 63.89 |
| Borehole (vs. piped) | -78.56 | 0.72 | -509.47 | 352.34 |
| Toilet facility used by household |  |  |  |  |
| Ventilated pit latrine (vs. flush toilet) | 100.45 | 0.74 | -485.35 | 686.25 |
| Pit latrine with roof (vs. flush toilet) | 245.33 | 0.39 | -312.93 | 803.59 |
| Traditional pit latrine (vs. flush toilet) | -96.18 | 0.77 | -740.18 | 547.81 |
| Total number of household assets | **244.90** | **0.01** | **64.78** | **425.02** |
| HFIAS score | **-192.52** | **0.01** | **-329.75** | **-55.29** |
| Total amount spent on food for household | 11.95 | 0.88 | -142.14 | 166.04 |
| Total amount spent on special foods for children <5 years | 205.71 | 0.10 | -42.42 | 453.83 |
| Purchase special foods for children  (vs. no) | **641.97** | **0.02** | **128.34** | **1155.61** |
| Mother’s body size preference for herself |  |  |  |  |
| Normal weight (vs. underweight) | -590.66 | 0.33 | -1785.83 | 604.51 |
| Overweight (vs. underweight) | -488.09 | 0.42 | -1672.66 | 696.47 |
| Obese (vs. underweight) | -485.90 | 0.43 | -1706.23 | 734.43 |
| Mother’s perception of healthy body size for herself |  |  |  |  |
| Overweight (vs. normal weight) | 65.92 | 0.82 | -498.14 | 629.98 |
| Obese (vs. normal weight) | 161.37 | 0.59 | -420.40 | 743.15 |
| No child cough in last 2 weeks (vs. yes) | -25.71 | 0.84 | -280.24 | 228.83 |
| No maternal fever in last 2 weeks (vs. yes) | -84.64 | 0.67 | -473.66 | 304.39 |
| Taste preference: grains | **281.86** | **0.02** | **48.75** | **514.97** |
| Taste preference: legumes | -166.71 | 0.20 | -422.92 | 89.49 |
| Taste preference: vegetables | -115.47 | 0.34 | -354.79 | 123.85 |
| Taste preference: fruit | 81.46 | 0.51 | -162.67 | 325.59 |
| Taste preference: meat and eggs | -154.28 | 0.19 | -386.59 | 78.03 |
| Taste preference: dairy | 45.19 | 0.60 | -124.76 | 215.15 |
| Taste preference: oil/fat | -52.04 | 0.61 | -254.61 | 150.52 |
| Taste preference: snacks | 17.14 | 0.82 | -129.46 | 163.74 |
| Taste preference: sweets | **422.08** | **0.01** | **126.56** | **717.60** |

| **Table 2.** Predictors of caloric intake among mothers in the rainy season. Model estimates from multivariate linear regression. P<0.05 are bold. | | | | |
| --- | --- | --- | --- | --- |
| **Variable** | **Coefficient** | **P-value** | **Lower 95% CI** | **Upper 95% CI** |
| Rural (vs. urban) | 146.55 | 0.61 | -419.69 | 712.79 |
| Main source of drinking water |  |  |  |  |
| Well (vs. piped) | -133.41 | 0.72 | -860.44 | 593.61 |
| Borehole (vs. piped) | -251.18 | 0.41 | -844.36 | 342.00 |
| Purchase special foods for children  (vs. no) | 81.26 | 0.69 | -325.97 | 488.49 |
| Mother’s body size preference for herself |  |  |  |  |
| Normal weight (vs. underweight) | -399.84 | 0.68 | -2303.11 | 1503.43 |
| Overweight (vs. underweight) | 10.10 | 0.99 | -1884.40 | 1904.59 |
| Obese (vs. underweight) | -62.35 | 0.95 | -2003.82 | 1879.12 |
| No child fever in last 2 weeks (vs. yes) | 164.21 | 0.33 | -167.74 | 496.16 |
| No maternal cough in last 2 weeks (vs. yes) | 326.16 | 0.09 | -47.43 | 699.75 |
| Total number of household assets | -51.86 | 0.67 | -294.13 | 190.40 |
| HFIAS score | **-265.33** | **0.01** | **-468.48** | **-62.18** |
| Total amount spent on food for household | -57.40 | 0.59 | -266.37 | 151.57 |
| How long it takes to get to nearest market/shop to purchase food | **-237.29** | **0.01** | **-405.80** | **-68.77** |
| Total amount spent on special foods for children <5 years | **392.84** | **0.00** | **167.19** | **618.49** |
| Taste preference: grains | -8.43 | 0.96 | -299.73 | 282.87 |
| Taste preference: tubers | 63.55 | 0.63 | -197.36 | 324.45 |
| Taste preference: vegetables | -292.66 | 0.11 | -646.88 | 61.55 |
| Taste preference: fruit | **352.48** | **0.04** | **18.87** | **686.08** |
| Taste preference: fish | 115.89 | 0.31 | -110.12 | 341.90 |
| Taste preference: oil/fat | 30.18 | 0.77 | -176.26 | 236.62 |
| Taste preference: snacks | 199.20 | 0.21 | -112.82 | 511.22 |
| Taste preference: sweets | -284.52 | 0.10 | -625.72 | 56.67 |

| **Table 3.** Predictors of caloric intake among children in the dry season. Model estimates from multivariate linear regression. P<0.05 are bold. | | | | |
| --- | --- | --- | --- | --- |
| **Variable** | **Coefficient** | **P-value** | **Lower 95% CI** | **Upper 95% CI** |
| Rural (vs. urban) | -118.25 | 0.45 | -422.74 | 186.24 |
| Mother secondary education or higher (vs. less) | 128.35 | 0.24 | -85.64 | 342.34 |
| Main source of drinking water |  |  |  |  |
| Well (vs. piped) | -234.28 | 0.20 | -590.22 | 121.66 |
| Borehole (vs. piped) | 39.78 | 0.81 | -280.94 | 360.50 |
| Toilet facility used by household |  |  |  |  |
| Ventilated pit latrine (vs. flush toilet) | 206.74 | 0.36 | -239.38 | 652.87 |
| Pit latrine with roof (vs. flush toilet) | 164.88 | 0.44 | -258.60 | 588.36 |
| Traditional pit latrine (vs. flush toilet) | 81.81 | 0.75 | -417.81 | 581.44 |
| Who purchases most food the family consumes |  |  |  |  |
| Husband/partner (vs. mother) | 101.42 | 0.44 | -158.22 | 361.05 |
| Both (vs. mother) | 252.14 | 0.13 | -78.42 | 582.70 |
| Other family member (vs. mother) | -38.55 | 0.84 | -420.97 | 343.88 |
| Purchase special foods for children  (vs. no) | 331.13 | 0.09 | -55.50 | 717.75 |
| No child diarrhea in last 2 weeks (vs. yes) | 75.37 | 0.47 | -130.82 | 281.55 |
| Age of mother | -7.31 | 0.88 | -103.95 | 89.34 |
| Age of child | **350.28** | **0.00** | **256.62** | **443.95** |
| Total number of household assets | 108.49 | 0.11 | -25.80 | 242.78 |
| HFIAS score | -39.36 | 0.45 | -141.54 | 62.81 |
| Total amount spent on food for household | 114.30 | 0.05 | -1.50 | 230.10 |
| Total amount spent on special foods for children <5 years | 56.20 | 0.56 | -132.70 | 245.11 |
| Taste preference: grains | 166.67 | 0.06 | -4.75 | 338.09 |
| Taste preference: legumes | 53.06 | 0.58 | -137.64 | 243.76 |
| Taste preference: vegetables | -137.00 | 0.11 | -303.71 | 29.71 |
| Taste preference: fruit | -41.26 | 0.65 | -222.42 | 139.91 |
| Taste preference: dairy | -101.33 | 0.12 | -228.32 | 25.66 |
| Taste preference: oil/fat | -40.70 | 0.60 | -192.94 | 111.53 |
| Taste preference: sweets | **264.58** | **0.01** | **53.17** | **475.99** |

| **Table 4.** Predictors of caloric intake among children in the rainy season. Model estimates from multivariate linear regression. P<0.05 are bold. | | | | |
| --- | --- | --- | --- | --- |
| **Variable** | **Coefficient** | **P-value** | **Lower 95% CI** | **Upper 95% CI** |
| Rural (vs. urban) | -152.27 | 0.40 | -505.91 | 201.36 |
| Mother secondary education or higher (vs. less) | 56.70 | 0.63 | -174.09 | 287.50 |
| Main source of drinking water |  |  |  |  |
| Well (vs. piped) | 84.68 | 0.72 | -377.20 | 546.55 |
| Borehole (vs. piped) | -5.08 | 0.98 | -382.37 | 372.22 |
| Purchase special foods for children  (vs. no) | -16.55 | 0.90 | -262.86 | 229.76 |
| No child diarrhea in last 2 weeks (vs. yes) | 25.28 | 0.84 | -215.72 | 266.28 |
| No child fever in last 2 weeks (vs. yes) | -57.88 | 0.60 | -276.45 | 160.69 |
| No child cough in last 2 weeks (vs. yes) | 196.53 | 0.10 | -41.08 | 434.15 |
| Age of child | **308.41** | **0.00** | **203.79** | **413.02** |
| Total number of household assets | 46.70 | 0.55 | -106.83 | 200.23 |
| HFIAS score | **-188.50** | **0.00** | **-316.14** | **-60.87** |
| Total amount spent on food for household | -22.15 | 0.74 | -152.10 | 107.80 |
| Total amount spent on special foods for children <5 years | **283.73** | **0.00** | **144.91** | **422.56** |
| Taste preference: grains | -33.20 | 0.72 | -216.44 | 150.03 |
| Taste preference: tubers | 108.77 | 0.20 | -58.41 | 275.96 |
| Taste preference: vegetables | -212.18 | 0.06 | -430.63 | 6.27 |
| Taste preference: fruit | 102.33 | 0.31 | -94.06 | 298.72 |
| Taste preference: fish | 96.97 | 0.18 | -46.17 | 240.12 |
| Taste preference: dairy | 29.76 | 0.70 | -122.56 | 182.07 |
| Taste preference: oil/fat | 112.82 | 0.09 | -16.43 | 242.07 |
| Taste preference: snacks | 67.99 | 0.50 | -129.26 | 265.24 |
| Taste preference: sweets | -110.66 | 0.35 | -344.99 | 123.67 |

| **Table 5.** Predictors of carbohydrate intake among mothers in the dry season. Model estimates from multivariate linear regression. P<0.05 are bold. | | | | |
| --- | --- | --- | --- | --- |
| **Variable** | **Coefficient** | **P-value** | **Lower 95% CI** | **Upper 95% CI** |
| Dyad Type |  |  |  |  |
| Overweight mother, normal weight child (vs. both overweight) | **-3.52** | **0.02** | **-6.50** | **-0.54** |
| Overweight child, normal weight mother (vs. both overweight) | -1.46 | 0.31 | -4.29 | 1.37 |
| Toilet facility used by household |  |  |  |  |
| Ventilated pit latrine (vs. flush toilet) | 1.53 | 0.56 | -3.66 | 6.72 |
| Pit latrine with roof (vs. flush toilet) | 2.11 | 0.40 | -2.77 | 7.00 |
| Traditional pit latrine (vs. flush toilet) | 2.28 | 0.44 | -3.54 | 8.09 |
| Who purchases most food the family consumes |  |  |  |  |
| Husband/partner (vs. mother) | 0.08 | 0.96 | -2.91 | 3.07 |
| Both (vs. mother) | -1.86 | 0.33 | -5.65 | 1.92 |
| Other family member (vs. mother) | 1.98 | 0.43 | -2.91 | 6.87 |
| Mother’s body size preference for her child |  |  |  |  |
| Normal weight (vs. underweight) | 1.24 | 0.74 | -6.09 | 8.57 |
| Overweight (vs. underweight) | 3.16 | 0.39 | -4.11 | 10.42 |
| Obese (vs. underweight) | 2.98 | 0.43 | -4.47 | 10.44 |
| No child diarrhea in last 2 weeks (vs. yes) | -0.85 | 0.49 | -3.28 | 1.58 |
| No mother diarrhea in last 2 weeks (vs. yes) | -1.38 | 0.43 | -4.81 | 2.04 |
| Age of child | -0.42 | 0.47 | -1.55 | 0.71 |
| Total number of household assets | -0.89 | 0.22 | -2.32 | 0.53 |
| HFIAS score | 0.14 | 0.82 | -1.04 | 1.31 |
| Female autonomy | -0.53 | 0.39 | -1.77 | 0.70 |
| Total amount spent on food for household | -0.39 | 0.54 | -1.64 | 0.85 |
| Total amount spent on special foods for children <5 years | -0.59 | 0.31 | -1.73 | 0.55 |
| Taste preference: fruit | 0.05 | 0.96 | -1.83 | 1.92 |
| Taste preference: meat and eggs | 0.29 | 0.77 | -1.61 | 2.18 |
| Taste preference: fish | 0.10 | 0.87 | -1.18 | 1.39 |
| Taste preference: snacks | 1.23 | 0.07 | -0.09 | 2.54 |
| Taste preference: sweets | -0.79 | 0.41 | -2.70 | 1.11 |

| **Table 6.** Predictors of carbohydrate intake among mothers in the rainy season. Model estimates from multivariate linear regression. P<0.05 are bold. | | | | |
| --- | --- | --- | --- | --- |
| **Variable** | **Coefficient** | **P-value** | **Lower 95% CI** | **Upper 95% CI** |
| Rural (vs. urban) | -1.12 | 0.47 | -4.20 | 1.95 |
| Mother secondary education or higher (vs. less) | -0.29 | 0.78 | -2.32 | 1.75 |
| Main source of drinking water |  |  |  |  |
| Well (vs. piped) | **4.15** | **0.04** | **0.17** | **8.13** |
| Borehole (vs. piped) | 1.18 | 0.47 | -2.06 | 4.42 |
| Toilet facility used by household |  |  |  |  |
| Ventilated pit latrine (vs. flush toilet) | 2.75 | 0.36 | -3.18 | 8.68 |
| Pit latrine with roof (vs. flush toilet) | 3.79 | 0.21 | -2.14 | 9.73 |
| Traditional pit latrine (vs. flush toilet) | 1.02 | 0.77 | -5.67 | 7.70 |
| Purchase special foods for children  (vs. no) | 0.64 | 0.57 | -1.55 | 2.82 |
| Mother’s body size preference for herself |  |  |  |  |
| Normal weight (vs. underweight) | -3.58 | 0.49 | -13.82 | 6.65 |
| Overweight (vs. underweight) | -2.77 | 0.59 | -12.99 | 7.45 |
| Obese (vs. underweight) | -1.90 | 0.72 | -12.20 | 8.40 |
| No child fever in last 2 weeks (vs. yes) | -1.18 | 0.20 | -2.99 | 0.63 |
| Household size | **1.09** | **0.01** | **0.22** | **1.96** |
| Total number of household assets | 0.00 | 1.00 | -1.37 | 1.37 |
| HFIAS score | 1.11 | 0.05 | 0.01 | 2.20 |
| Female autonomy | -0.23 | 0.63 | -1.15 | 0.69 |
| Total amount spent on food for household | -0.88 | 0.13 | -2.02 | 0.27 |
| How long it takes to get to nearest market/shop to purchase food | 0.44 | 0.34 | -0.46 | 1.35 |
| Total amount spent on special foods for children <5 years | -0.64 | 0.31 | -1.87 | 0.59 |
| Taste preference: dairy | **-1.58** | **0.02** | **-2.87** | **-0.29** |
| Taste preference: oil/fat | -0.95 | 0.09 | -2.06 | 0.16 |
| Taste preference: sweets | 1.06 | 0.13 | -0.33 | 2.44 |

| **Table 7.** Predictors of carbohydrate intake among children in the dry season. Model estimates from multivariate linear regression. P<0.05 are bold. | | | | |
| --- | --- | --- | --- | --- |
| **Variable** | **Coefficient** | **P-value** | **Lower 95% CI** | **Upper 95% CI** |
| Dyad Type |  |  |  |  |
| Overweight mother, normal weight child (vs. both overweight) | -3.23 | 0.07 | -6.68 | 0.22 |
| Overweight child, normal weight mother (vs. both overweight) | -1.87 | 0.30 | -5.45 | 1.71 |
| Toilet facility used by household |  |  |  |  |
| Ventilated pit latrine (vs. flush toilet) | **-9.09** | **0.01** | **-15.44** | **-2.74** |
| Pit latrine with roof (vs. flush toilet) | **-7.95** | **0.01** | **-13.80** | **-2.11** |
| Traditional pit latrine (vs. flush toilet) | **-8.32** | **0.02** | **-15.09** | **-1.56** |
| Purchase special foods for children  (vs. no) | 3.22 | 0.24 | -2.18 | 8.63 |
| No mother diarrhea in last 2 weeks (vs. yes) | -3.73 | 0.08 | -7.90 | 0.44 |
| HFIAS score | 0.77 | 0.30 | -0.68 | 2.23 |
| Total amount spent on food for household | -1.18 | 0.12 | -2.68 | 0.32 |
| Total amount spent on special foods for children <5 years | -0.03 | 0.98 | -2.65 | 2.59 |
| Taste preference: legumes | **-2.23** | **0.00** | **-3.72** | **-0.75** |
| Taste preference: fish | 1.28 | 0.10 | -0.26 | 2.83 |

| **Table 8.** Predictors of carbohydrate intake among children in the rainy season. Model estimates from multivariate linear regression. P<0.05 are bold. | | | | |
| --- | --- | --- | --- | --- |
| **Variable** | **Coefficient** | **P-value** | **Lower 95% CI** | **Upper 95% CI** |
| Rural (vs. urban) | 0.22 | 0.91 | -3.78 | 4.22 |
| Main source of drinking water |  |  |  |  |
| Well (vs. piped) | **5.36** | **0.05** | **0.10** | **10.61** |
| Borehole (vs. piped) | 0.63 | 0.77 | -3.63 | 4.89 |
| Mother’s body size preference for herself |  |  |  |  |
| Normal weight (vs. underweight) | 0.47 | 0.94 | -12.15 | 13.09 |
| Overweight (vs. underweight) | -2.05 | 0.75 | -14.57 | 10.46 |
| Obese (vs. underweight) | -0.51 | 0.94 | -13.28 | 12.26 |
| Mother’s perception of healthy body size for herself |  |  |  |  |
| Overweight (vs. normal weight) | 0.64 | 0.83 | -5.10 | 6.37 |
| Obese (vs. normal weight) | 2.30 | 0.43 | -3.45 | 8.06 |
| No child fever in last 2 weeks (vs. yes) | -1.08 | 0.37 | -3.43 | 1.28 |
| Age of child | **1.42** | **0.02** | **0.24** | **2.60** |
| Total number of household assets | 0.18 | 0.83 | -1.48 | 1.84 |
| HFIAS score | 0.74 | 0.28 | -0.61 | 2.08 |
| Total amount spent on food for household | -0.72 | 0.32 | -2.15 | 0.70 |
| How long it takes to get to nearest market/shop to purchase food | 0.33 | 0.58 | -0.84 | 1.49 |
| Total amount spent on special foods for children <5 years | 0.38 | 0.58 | -0.96 | 1.72 |
| Taste preference: dairy | -0.42 | 0.53 | -1.74 | 0.90 |
| Taste preference: oil/fat | -1.11 | 0.10 | -2.42 | 0.20 |

| **Table 9.** Predictors of fat intake among mothers in the dry season. Model estimates from multivariate linear regression. P<0.05 are bold. | | | | |
| --- | --- | --- | --- | --- |
| **Variable** | **Coefficient** | **P-value** | **Lower 95% CI** | **Upper 95% CI** |
| Dyad Type |  |  |  |  |
| Overweight mother, normal weight child (vs. both overweight) | **4.11** | **0.01** | **1.24** | **6.98** |
| Overweight child, normal weight mother (vs. both overweight) | 1.44 | 0.32 | -1.40 | 4.29 |
| Rural (vs. urban) | -0.98 | 0.45 | -3.50 | 1.55 |
| Mother secondary education or higher (vs. less) | -0.58 | 0.64 | -3.00 | 1.85 |
| Toilet facility used by household |  |  |  |  |
| Ventilated pit latrine (vs. flush toilet) | -1.49 | 0.56 | -6.55 | 3.58 |
| Pit latrine with roof (vs. flush toilet) | -2.07 | 0.40 | -6.86 | 2.72 |
| Traditional pit latrine (vs. flush toilet) | -2.01 | 0.48 | -7.63 | 3.61 |
| No child diarrhea in last 2 weeks (vs. yes) | 1.71 | 0.16 | -0.68 | 4.11 |
| Total number of household assets | 0.93 | 0.23 | -0.58 | 2.44 |
| HFIAS score | -0.59 | 0.33 | -1.78 | 0.60 |
| Female autonomy | 0.54 | 0.33 | -0.54 | 1.63 |
| Total amount spent on food for household | 0.39 | 0.55 | -0.90 | 1.69 |
| How long it takes to get to nearest market/shop to purchase food | -0.73 | 0.20 | -1.84 | 0.38 |
| Total amount spent on special foods for children <5 years | 0.43 | 0.46 | -0.71 | 1.56 |
| Taste preference: fruit | 0.38 | 0.69 | -1.48 | 2.25 |
| Taste preference: meat and eggs | -0.28 | 0.78 | -2.19 | 1.63 |
| Taste preference: fish | 0.07 | 0.92 | -1.26 | 1.40 |
| Taste preference: dairy | -0.63 | 0.35 | -1.95 | 0.68 |
| Taste preference: oil/fat | 0.85 | 0.34 | -0.90 | 2.59 |
| Taste preference: snacks | -1.28 | 0.06 | -2.64 | 0.07 |
| Taste preference: sweets | -0.39 | 0.74 | -2.75 | 1.96 |

| **Table 10.** Predictors of fat intake among mothers in the rainy season. Model estimates from multivariate linear regression. P<0.05 are bold. | | | | |
| --- | --- | --- | --- | --- |
| **Variable** | **Coefficient** | **P-value** | **Lower 95% CI** | **Upper 95% CI** |
| Rural (vs. urban) | 0.33 | 0.82 | -2.53 | 3.18 |
| Mother secondary education or higher (vs. less) | 0.73 | 0.45 | -1.17 | 2.62 |
| Main source of drinking water |  |  |  |  |
| Well (vs. piped) | **-3.86** | **0.04** | **-7.57** | **-0.15** |
| Borehole (vs. piped) | -1.45 | 0.34 | -4.45 | 1.55 |
| Toilet facility used by household |  |  |  |  |
| Ventilated pit latrine (vs. flush toilet) | -2.36 | 0.40 | -7.84 | 3.13 |
| Pit latrine with roof (vs. flush toilet) | -3.48 | 0.21 | -8.97 | 2.01 |
| Traditional pit latrine (vs. flush toilet) | -1.10 | 0.73 | -7.30 | 5.09 |
| Purchase special foods for children  (vs. no) | -0.93 | 0.37 | -2.96 | 1.10 |
| Mother’s body size preference for herself |  |  |  |  |
| Normal weight (vs. underweight) | 2.63 | 0.58 | -6.82 | 12.08 |
| Overweight (vs. underweight) | 2.24 | 0.64 | -7.16 | 11.64 |
| Obese (vs. underweight) | 1.41 | 0.77 | -8.09 | 10.90 |
| Mother’s perception of healthy body size for herself |  |  |  |  |
| Overweight (vs. normal weight) | -1.46 | 0.48 | -5.54 | 2.62 |
| Obese (vs. normal weight) | -0.71 | 0.73 | -4.85 | 3.42 |
| No child fever in last 2 weeks (vs. yes) | 0.89 | 0.30 | -0.81 | 2.59 |
| Household size | **-1.13** | **0.01** | **-1.93** | **-0.33** |
| Total number of household assets | 0.24 | 0.71 | -1.03 | 1.52 |
| HFIAS score | **-1.46** | **0.01** | **-2.48** | **-0.44** |
| Female autonomy | 0.19 | 0.66 | -0.67 | 1.05 |
| Total amount spent on food for household | 0.82 | 0.13 | -0.24 | 1.88 |
| How long it takes to get to nearest market/shop to purchase food | -0.59 | 0.17 | -1.44 | 0.26 |
| Total amount spent on special foods for children <5 years | 0.52 | 0.38 | -0.63 | 1.66 |
| Taste preference: dairy | 0.78 | 0.12 | -0.19 | 1.74 |
| Taste preference: oil/fat | 0.47 | 0.33 | -0.47 | 1.41 |

| **Table 11.** Predictors of fat intake among children in the dry season. Model estimates from multivariate linear regression. P<0.05 are bold. | | | | |
| --- | --- | --- | --- | --- |
| **Variable** | **Coefficient** | **P-value** | **Lower 95% CI** | **Upper 95% CI** |
| Dyad Type |  |  |  |  |
| Overweight mother, normal weight child (vs. both overweight) | 2.88 | 0.11 | -0.65 | 6.41 |
| Overweight child, normal weight mother (vs. both overweight) | 0.78 | 0.67 | -2.84 | 4.39 |
| Rural (vs. urban) | -0.18 | 0.91 | -3.39 | 3.03 |
| Mother secondary education or higher (vs. less) | 0.10 | 0.95 | -3.00 | 3.19 |
| Toilet facility used by household |  |  |  |  |
| Ventilated pit latrine (vs. flush toilet) | **10.20** | **0.00** | **3.71** | **16.69** |
| Pit latrine with roof (vs. flush toilet) | **8.32** | **0.01** | **2.14** | **14.50** |
| Traditional pit latrine (vs. flush toilet) | **9.02** | **0.02** | **1.74** | **16.29** |
| No mother diarrhea in last 2 weeks (vs. yes) | 3.42 | 0.11 | -0.82 | 7.66 |
| Total number of household assets | 0.69 | 0.48 | -1.24 | 2.61 |
| HFIAS score | -1.25 | 0.11 | -2.78 | 0.27 |
| Total amount spent on food for household | 0.74 | 0.36 | -0.86 | 2.34 |
| How long it takes to get to nearest market/shop to purchase food | -0.79 | 0.27 | -2.20 | 0.61 |
| Total amount spent on special foods for children <5 years | 0.95 | 0.18 | -0.45 | 2.35 |
| Taste preference: fish | -0.87 | 0.24 | -2.33 | 0.59 |

| **Table 12.** Predictors of fat intake among children in the rainy season. Model estimates from multivariate linear regression. P<0.05 are bold. | | | | |
| --- | --- | --- | --- | --- |
| **Variable** | **Coefficient** | **P-value** | **Lower 95% CI** | **Upper 95% CI** |
| Rural (vs. urban) | -1.36 | 0.51 | -5.37 | 2.66 |
| Mother secondary education or higher (vs. less) | -0.24 | 0.86 | -2.87 | 2.40 |
| Main source of drinking water |  |  |  |  |
| Well (vs. piped) | -4.90 | 0.07 | -10.17 | 0.38 |
| Borehole (vs. piped) | -1.13 | 0.60 | -5.40 | 3.14 |
| Toilet facility used by household |  |  |  |  |
| Ventilated pit latrine (vs. flush toilet) | -2.97 | 0.44 | -10.60 | 4.66 |
| Pit latrine with roof (vs. flush toilet) | -2.16 | 0.58 | -9.82 | 5.51 |
| Traditional pit latrine (vs. flush toilet) | -1.76 | 0.69 | -10.43 | 6.91 |
| Mother’s body size preference for herself |  |  |  |  |
| Normal weight (vs. underweight) | 1.01 | 0.88 | -11.87 | 13.88 |
| Overweight (vs. underweight) | 3.21 | 0.62 | -9.61 | 16.03 |
| Obese (vs. underweight) | 1.79 | 0.79 | -11.21 | 14.78 |
| Mother’s perception of healthy body size for herself |  |  |  |  |
| Overweight (vs. normal weight) | -0.67 | 0.82 | -6.40 | 5.05 |
| Obese (vs. normal weight) | -2.55 | 0.39 | -8.33 | 3.22 |
| Age of child | **-1.57** | **0.01** | **-2.76** | **-0.38** |
| Number of children <5 years in household | -0.71 | 0.22 | -1.85 | 0.43 |
| Total number of household assets | 0.28 | 0.76 | -1.49 | 2.05 |
| HFIAS score | -1.22 | 0.08 | -2.56 | 0.12 |
| Total amount spent on food for household | 0.55 | 0.46 | -0.92 | 2.01 |
| How long it takes to get to nearest market/shop to purchase food | -0.44 | 0.47 | -1.62 | 0.74 |
| Total amount spent on special foods for children <5 years | -0.55 | 0.42 | -1.89 | 0.79 |
| Taste preference: dairy | 0.30 | 0.65 | -1.01 | 1.62 |
| Taste preference: oil/fat | 0.97 | 0.15 | -0.35 | 2.29 |

| **Table 13.** Predictors of protein intake among mothers in the dry season. Model estimates from multivariate linear regression. P<0.05 are bold. | | | | |
| --- | --- | --- | --- | --- |
| **Variable** | **Coefficient** | **P-value** | **Lower 95% CI** | **Upper 95% CI** |
| Rural (vs. urban) | **1.12** | **0.03** | **0.14** | **2.10** |
| Main source of drinking water |  |  |  |  |
| Well (vs. piped) | -0.12 | 0.84 | -1.27 | 1.03 |
| Borehole (vs. piped) | -0.18 | 0.73 | -1.23 | 0.86 |
| Who purchases most food the family consumes |  |  |  |  |
| Husband/partner (vs. mother) | -0.80 | 0.05 | -1.60 | 0.00 |
| Both (vs. mother) | 0.32 | 0.55 | -0.73 | 1.37 |
| Other family member (vs. mother) | -1.17 | 0.06 | -2.37 | 0.03 |
| Purchase special foods for children  (vs. no) | -0.55 | 0.10 | -1.20 | 0.11 |
| Mother’s body size preference for herself |  |  |  |  |
| Normal weight (vs. underweight) | 2.26 | 0.12 | -0.57 | 5.08 |
| Overweight (vs. underweight) | 1.68 | 0.24 | -1.12 | 4.49 |
| Obese (vs. underweight) | 2.40 | 0.10 | -0.49 | 5.29 |
| Total number of household assets | 0.02 | 0.92 | -0.38 | 0.42 |
| HFIAS score | 0.32 | 0.06 | -0.01 | 0.66 |
| Total amount spent on food for household | -0.01 | 0.96 | -0.36 | 0.35 |
| How long it takes to get to nearest market/shop to purchase food | 0.31 | 0.05 | 0.00 | 0.62 |
| Taste preference: fish | -0.09 | 0.59 | -0.43 | 0.24 |
| Taste preference: oil/fat | 0.46 | 0.06 | -0.02 | 0.93 |
| Taste preference: snacks | 0.10 | 0.57 | -0.24 | 0.44 |
| Taste preference: sweets | 0.03 | 0.90 | -0.47 | 0.54 |

| **Table 14.** Predictors of protein intake among mothers in the rainy season. Model estimates from multivariate linear regression. P<0.05 are bold. | | | | |
| --- | --- | --- | --- | --- |
| **Variable** | **Coefficient** | **P-value** | **Lower 95% CI** | **Upper 95% CI** |
| Female sex of child (vs. male) |  |  |  |  |
| Female | 0.08 | 0.76 | -0.44 | 0.60 |
| Kasungu district (vs. Lilongwe) | 0.38 | 0.15 | -0.14 | 0.91 |
| Rural (vs. urban) | 0.80 | 0.09 | -0.11 | 1.70 |
| Mother secondary education or higher (vs. less) | -0.27 | 0.36 | -0.86 | 0.31 |
| Main source of drinking water |  |  |  |  |
| Well (vs. piped) | -0.27 | 0.66 | -1.48 | 0.93 |
| Borehole (vs. piped) | 0.09 | 0.85 | -0.88 | 1.07 |
| Mother’s perception of healthy body size for herself |  |  |  |  |
| Overweight (vs. normal weight) | -0.21 | 0.74 | -1.45 | 1.03 |
| Obese (vs. normal weight) | -0.10 | 0.88 | -1.34 | 1.15 |
| No mother cough in last 2 weeks (vs. yes) | -0.58 | 0.06 | -1.18 | 0.02 |
| Age of child | **0.30** | **0.03** | **0.04** | **0.57** |
| Total number of household assets | -0.09 | 0.19 | -0.46 | 0.29 |
| HFIAS score | 0.19 | 0.23 | -0.12 | 0.49 |
| Total amount spent on food for household | 0.07 | 0.16 | -0.25 | 0.38 |
| Taste preference: dairy | 0.31 | 0.14 | 0.04 | 0.58 |

| **Table 15.** Predictors of protein intake among children in the dry season. Model estimates from multivariate linear regression. P<0.05 are bold. | | | | |
| --- | --- | --- | --- | --- |
| **Variable** | **Coefficient** | **P-value** | **Lower 95% CI** | **Upper 95% CI** |
| Dyad Type |  |  |  |  |
| Overweight mother, normal weight child (vs. both overweight) | -0.05 | 0.92 | -1.09 | 0.99 |
| Overweight child, normal weight mother (vs. both overweight) | 0.49 | 0.35 | -0.54 | 1.53 |
| Rural (vs. urban) | 0.36 | 0.59 | -0.95 | 1.66 |
| Main source of drinking water |  |  |  |  |
| Well (vs. piped) | 0.14 | 0.86 | -1.40 | 1.68 |
| Borehole (vs. piped) | 0.64 | 0.36 | -0.73 | 2.01 |
| Toilet facility used by household |  |  |  |  |
| Ventilated pit latrine (vs. flush toilet) | 0.02 | 0.98 | -1.83 | 1.87 |
| Pit latrine with roof (vs. flush toilet) | 0.37 | 0.68 | -1.39 | 2.14 |
| Traditional pit latrine (vs. flush toilet) | -0.46 | 0.66 | -2.51 | 1.58 |
| Mother’s body size preference for herself |  |  |  |  |
| Normal weight (vs. underweight) | 2.28 | 0.24 | -1.52 | 6.08 |
| Overweight (vs. underweight) | 1.16 | 0.55 | -2.62 | 4.93 |
| Obese (vs. underweight) | 2.30 | 0.25 | -1.59 | 6.18 |
| Age of child | 0.33 | 0.12 | -0.08 | 0.75 |
| Total number of household assets | -0.06 | 0.82 | -0.60 | 0.48 |
| HFIAS score | **0.49** | **0.03** | **0.05** | **0.94** |
| Total amount spent on food for household | -0.10 | 0.68 | -0.57 | 0.37 |
| How long it takes to get to nearest market/shop to purchase food | **0.48** | **0.02** | **0.08** | **0.87** |
| Taste preference: grains | -0.28 | 0.42 | -0.97 | 0.40 |
| Taste preference: vegetables | 0.25 | 0.52 | -0.51 | 1.01 |
| Taste preference: fruit | 0.44 | 0.26 | -0.33 | 1.21 |
| Taste preference: meat and eggs | 0.23 | 0.53 | -0.49 | 0.96 |
| Taste preference: fish | 0.24 | 0.31 | -0.22 | 0.71 |
| Taste preference: oil/fat | 0.30 | 0.36 | -0.34 | 0.94 |
| Taste preference: sweets | -0.30 | 0.52 | -1.22 | 0.63 |

| **Table 16.** Predictors of protein intake among children in the rainy season. Model estimates from multivariate linear regression. P<0.05 are bold. | | | | |
| --- | --- | --- | --- | --- |
| **Variable** | **Coefficient** | **P-value** | **Lower 95% CI** | **Upper 95% CI** |
| Female sex of child (vs. male) | **0.63** | **0.04** | **0.02** | **1.24** |
| Rural (vs. urban) | **1.14** | **0.04** | **0.05** | **2.22** |
| Mother secondary education or higher (vs. less) | -0.23 | 0.51 | -0.94 | 0.47 |
| Main source of drinking water |  |  |  |  |
| Well (vs. piped) | -1.10 | 0.13 | -2.50 | 0.31 |
| Borehole (vs. piped) | -0.08 | 0.90 | -1.22 | 1.06 |
| Toilet facility used by household |  |  |  |  |
| Ventilated pit latrine (vs. flush toilet) | -0.77 | 0.45 | -2.80 | 1.25 |
| Pit latrine with roof (vs. flush toilet) | -0.20 | 0.85 | -2.25 | 1.84 |
| Traditional pit latrine (vs. flush toilet) | -0.48 | 0.68 | -2.78 | 1.82 |
| Mother’s body size preference for herself |  |  |  |  |
| Normal weight (vs. underweight) | 0.78 | 0.66 | -2.74 | 4.31 |
| Overweight (vs. underweight) | 0.99 | 0.58 | -2.51 | 4.48 |
| Obese (vs. underweight) | 0.79 | 0.66 | -2.78 | 4.36 |
| Mother’s perception of healthy body size for herself |  |  |  |  |
| Overweight (vs. normal weight) | -0.32 | 0.67 | -1.84 | 1.19 |
| Obese (vs. normal weight) | -0.29 | 0.72 | -1.87 | 1.29 |
| Mother’s body size preference for her child |  |  |  |  |
| Normal weight (vs. underweight) | 1.37 | 0.27 | -1.05 | 3.80 |
| Overweight (vs. underweight) | 1.70 | 0.17 | -0.75 | 4.16 |
| Obese (vs. underweight) | 1.16 | 0.36 | -1.34 | 3.66 |
| No mother cough in last 2 weeks (vs. yes) | **-0.71** | **0.05** | **-1.41** | **-0.01** |
| Age of child | **0.34** | **0.04** | **0.02** | **0.66** |
| Number of children <5 years in household | 0.28 | 0.07 | -0.03 | 0.58 |
| Total number of household assets | -0.25 | 0.29 | -0.72 | 0.22 |
| HFIAS score | **0.47** | **0.01** | **0.12** | **0.83** |
| Total amount spent on food for household | 0.01 | 0.96 | -0.38 | 0.40 |
| Total amount spent on special foods for children <5 years | 0.15 | 0.39 | -0.20 | 0.51 |
| Taste preference: grains | **0.36** | **0.05** | **0.00** | **0.72** |
| Taste preference: meat and eggs | 0.16 | 0.41 | -0.22 | 0.53 |

| **Table 17.** Predictors of grain intake among mothers in the dry season. Model estimates from multivariate linear regression. P<0.05 are bold. | | | | |
| --- | --- | --- | --- | --- |
| **Variable** | **Coefficient** | **P-value** | **Lower 95% CI** | **Upper 95% CI** |
| Dyad Type |  |  |  |  |
| Overweight mother, normal weight child (vs. both overweight) | **-151.60** | **0.02** | **-275.42** | **-27.78** |
| Overweight child, normal weight mother (vs. both overweight) | 58.07 | 0.37 | -70.42 | 186.56 |
| Kasungu district (vs. Lilongwe) | 83.50 | 0.09 | -13.71 | 180.72 |
| Main source of drinking water |  |  |  |  |
| Well (vs. piped) | -95.33 | 0.26 | -259.93 | 69.27 |
| Borehole (vs. piped) | -42.17 | 0.49 | -162.50 | 78.16 |
| Toilet facility used by household |  |  |  |  |
| Ventilated pit latrine (vs. flush toilet) | 157.43 | 0.19 | -78.21 | 393.08 |
| Pit latrine with roof (vs. flush toilet) | 210.33 | 0.07 | -16.96 | 437.63 |
| Traditional pit latrine (vs. flush toilet) | 99.91 | 0.46 | -166.67 | 366.50 |
| Who purchases most food the family consumes |  |  |  |  |
| Husband/partner (vs. mother) | 109.61 | 0.11 | -24.10 | 243.32 |
| Both (vs. mother) | 12.54 | 0.89 | -162.32 | 187.40 |
| Other family member (vs. mother) | 59.14 | 0.56 | -140.02 | 258.30 |
| Purchase special foods for children  (vs. no) | -18.46 | 0.86 | -218.50 | 181.58 |
| Mother’s body size preference for herself |  |  |  |  |
| Normal weight (vs. underweight) | **-481.74** | **0.05** | **-953.09** | **-10.38** |
| Overweight (vs. underweight) | -410.49 | 0.08 | -874.78 | 53.80 |
| Obese (vs. underweight) | -477.93 | 0.05 | -959.53 | 3.66 |
| Mother’s body size preference for her child |  |  |  |  |
| Normal weight (vs. underweight) | 57.28 | 0.74 | -276.29 | 390.84 |
| Overweight (vs. underweight) | 175.28 | 0.30 | -159.89 | 510.45 |
| Obese (vs. underweight) | 191.22 | 0.28 | -156.58 | 539.03 |
| No child fever in last 2 weeks (vs. yes) | 68.01 | 0.18 | -31.94 | 167.96 |
| Total number of household assets | 59.15 | 0.08 | -7.79 | 126.09 |
| HFIAS score | -33.01 | 0.24 | -88.53 | 22.52 |
| Total amount spent on special foods for children <5 years | -22.96 | 0.64 | -118.29 | 72.37 |
| Taste preference: grains | **133.43** | **0.00** | **83.65** | **183.21** |

| **Table 18.** Predictors of grain intake among mothers in the rainy season. Model estimates from multivariate linear regression. P<0.05 are bold. | | | | |
| --- | --- | --- | --- | --- |
| **Variable** | **Coefficient** | **P-value** | **Lower 95% CI** | **Upper 95% CI** |
| Dyad Type |  |  |  |  |
| Overweight mother, normal weight child (vs. both overweight) | **-235.39** | **0.01** | **-403.84** | **-66.94** |
| Overweight child, normal weight mother (vs. both overweight) | -16.45 | 0.86 | -197.86 | 164.95 |
| Mother secondary education or higher (vs. less) | -88.26 | 0.27 | -244.84 | 68.33 |
| Toilet facility used by household |  |  |  |  |
| Ventilated pit latrine (vs. flush toilet) | 315.25 | 0.18 | -141.90 | 772.40 |
| Pit latrine with roof (vs. flush toilet) | 424.66 | 0.06 | -23.24 | 872.57 |
| Traditional pit latrine (vs. flush toilet) | 425.17 | 0.09 | -73.16 | 923.50 |
| Who purchases most food the family consumes |  |  |  |  |
| Husband/partner (vs. mother) | -65.01 | 0.54 | -273.46 | 143.43 |
| Both (vs. mother) | -166.69 | 0.20 | -421.83 | 88.44 |
| Other family member (vs. mother) | -234.47 | 0.15 | -553.03 | 84.09 |
| Purchase special foods for children  (vs. no) | 123.70 | 0.13 | -38.35 | 285.76 |
| Mother’s body size preference for herself |  |  |  |  |
| Normal weight (vs. underweight) | **-1145.07** | **0.01** | **-1948.30** | **-341.84** |
| Overweight (vs. underweight) | **-1046.61** | **0.01** | **-1844.12** | **-249.09** |
| Obese (vs. underweight) | **-1114.94** | **0.01** | **-1932.29** | **-297.58** |
| Female autonomy | -50.44 | 0.20 | -128.09 | 27.20 |
| How long it takes to get to nearest market/shop to purchase food | **-79.40** | **0.03** | **-148.85** | **-9.95** |
| Total amount spent on special foods for children <5 years | 68.14 | 0.13 | -19.90 | 156.17 |
| Taste preference: grains | **82.29** | **0.02** | **10.85** | **153.72** |

| **Table 19.** Predictors of grain intake among children in the dry season. Model estimates from multivariate linear regression. P<0.05 are bold. | | | | |
| --- | --- | --- | --- | --- |
| **Variable** | **Coefficient** | **P-value** | **Lower 95% CI** | **Upper 95% CI** |
| Toilet facility used by household |  |  |  |  |
| Ventilated pit latrine (vs. flush toilet) | 120.90 | 0.05 | -2.08 | 243.89 |
| Pit latrine with roof (vs. flush toilet) | 60.43 | 0.29 | -51.50 | 172.35 |
| Traditional pit latrine (vs. flush toilet) | 72.97 | 0.26 | -53.20 | 199.15 |
| Purchase special foods for children  (vs. no) | 14.23 | 0.78 | -86.14 | 114.60 |
| Age of mother | 8.57 | 0.56 | -20.08 | 37.22 |
| Age of child | **72.63** | **0.00** | **46.01** | **99.25** |
| Household size | 5.17 | 0.72 | -23.63 | 33.97 |
| Number of children <5 years in household | 23.84 | 0.07 | -2.10 | 49.78 |
| Total amount spent on special foods for children <5 years | -28.98 | 0.26 | -79.11 | 21.15 |
| Taste preference: grains | **64.69** | **0.00** | **38.16** | **91.23** |

| **Table 20.** Predictors of grain intake among children in the rainy season. Model estimates from multivariate linear regression. P<0.05 are bold. | | | | |
| --- | --- | --- | --- | --- |
| **Variable** | **Coefficient** | **P-value** | **Lower 95% CI** | **Upper 95% CI** |
| Mother secondary education or higher (vs. less) | 16.11 | 0.67 | -58.81 | 91.04 |
| Main source of drinking water |  |  |  |  |
| Well (vs. piped) | 95.51 | 0.14 | -30.23 | 221.24 |
| Borehole (vs. piped) | 20.06 | 0.63 | -62.67 | 102.80 |
| Age of child | **114.12** | **0.00** | **80.96** | **147.29** |
| Total number of household assets | -23.84 | 0.26 | -65.03 | 17.34 |
| Female autonomy | -32.72 | 0.05 | -65.99 | 0.55 |
| Taste preference: grains | **70.13** | **0.00** | **37.27** | **103.00** |

| **Table 21.** Predictors of tubers intake among mothers in the dry season. Model estimates from multivariate linear regression. P<0.05 are bold. | | | | |
| --- | --- | --- | --- | --- |
| **Variable** | **Coefficient** | **P-value** | **Lower 95% CI** | **Upper 95% CI** |
| Total number of household assets | 1.17 | 0.89 | -16.03 | 18.36 |
| How long it takes to get to nearest market/shop to purchase food | -12.59 | 0.15 | -29.79 | 4.61 |

| **Table 22.** Predictors of tubers intake among mothers in the rainy season. Model estimates from multivariate linear regression. P<0.05 are bold. | | | | |
| --- | --- | --- | --- | --- |
| **Variable** | **Coefficient** | **P-value** | **Lower 95% CI** | **Upper 95% CI** |
| Kasungu district (vs. Lilongwe) | 27.74 | 0.07 | -2.63 | 58.11 |
| Rural (vs. urban) | -49.31 | 0.06 | -101.49 | 2.86 |
| Mother secondary education or higher (vs. less) | -16.98 | 0.32 | -50.65 | 16.70 |
| Main source of drinking water |  |  |  |  |
| Well (vs. piped) | -14.56 | 0.67 | -82.69 | 53.57 |
| Borehole (vs. piped) | -17.38 | 0.54 | -72.85 | 38.09 |
| Purchase special foods for children  (vs. no) | 24.40 | 0.19 | -11.80 | 60.59 |
| Total number of household assets | 0.34 | 0.98 | -21.93 | 22.61 |
| HFIAS score | -3.64 | 0.70 | -22.17 | 14.89 |
| Total amount spent on food for household | -5.24 | 0.58 | -24.08 | 13.59 |
| Total amount spent on special foods for children <5 years | 4.34 | 0.68 | -16.29 | 24.96 |
| Taste preference: tubers | 3.15 | 0.69 | -12.42 | 18.72 |

| **Table 23.** Predictors of tubers intake among children in the dry season. Model estimates from multivariate linear regression. P<0.05 are bold. | | | | |
| --- | --- | --- | --- | --- |
| **Variable** | **Coefficient** | **P-value** | **Lower 95% CI** | **Upper 95% CI** |
| Dyad Type |  |  |  |  |
| Overweight mother, normal weight child (vs. both overweight) | 3.98 | 0.62 | -11.98 | 19.94 |
| Overweight child, normal weight mother (vs. both overweight) | 0.99 | 0.91 | -15.99 | 17.98 |
| No mother diarrhea in last 2 weeks (vs. yes) | **-25.67** | **0.01** | **-45.69** | **-5.64** |
| Age of child | **9.92** | **0.00** | **3.16** | **16.67** |
| Total amount spent on special foods for children <5 years | -6.33 | 0.05 | -12.78 | 0.12 |

| **Table 24.** Predictors of tubers intake among children in the rainy season. Model estimates from multivariate linear regression. P<0.05 are bold. | | | | |
| --- | --- | --- | --- | --- |
| **Variable** | **Coefficient** | **P-value** | **Lower 95% CI** | **Upper 95% CI** |
| Rural (vs. urban) | -17.57 | 0.18 | -43.21 | 8.06 |
| Mother secondary education or higher (vs. less) | -8.88 | 0.30 | -25.58 | 7.83 |
| Main source of drinking water |  |  |  |  |
| Well (vs. piped) | 9.60 | 0.58 | -24.18 | 43.38 |
| Borehole (vs. piped) | -12.93 | 0.36 | -40.71 | 14.86 |
| Purchase special foods for children  (vs. no) | **21.65** | **0.02** | **3.69** | **39.61** |
| No child fever in last 2 weeks (vs. yes) | -6.07 | 0.44 | -21.39 | 9.25 |
| No mother cough in last 2 weeks (vs. yes) | 13.16 | 0.13 | -4.05 | 30.38 |
| Age of mother | 6.37 | 0.11 | -1.52 | 14.27 |
| Age of child | 4.67 | 0.23 | -3.04 | 12.38 |
| Total number of household assets | 0.93 | 0.87 | -10.15 | 12.02 |
| HFIAS score | -1.24 | 0.79 | -10.41 | 7.94 |
| Total amount spent on food for household | -2.84 | 0.55 | -12.20 | 6.53 |
| Total amount spent on special foods for children <5 years | -1.61 | 0.75 | -11.74 | 8.51 |
| Taste preference: tubers | 7.30 | 0.07 | -0.46 | 15.06 |

| **Table 25.** Predictors of legumes intake among mothers in the dry season. Model estimates from multivariate linear regression. P<0.05 are bold. | | | | |
| --- | --- | --- | --- | --- |
| **Variable** | **Coefficient** | **P-value** | **Lower 95% CI** | **Upper 95% CI** |
| Rural (vs. urban) | -35.86 | 0.26 | -97.79 | 26.07 |
| Main source of drinking water |  |  |  |  |
| Well (vs. piped) | -69.91 | 0.06 | -142.35 | 2.53 |
| Borehole (vs. piped) | -0.52 | 0.99 | -66.57 | 65.54 |
| Purchase special foods for children  (vs. no) | -7.96 | 0.84 | -86.12 | 70.21 |
| Mother’s body size preference for herself |  |  |  |  |
| Normal weight (vs. underweight) | 156.05 | 0.08 | -21.12 | 333.22 |
| Overweight (vs. underweight) | 116.98 | 0.19 | -58.97 | 292.94 |
| Obese (vs. underweight) | 140.50 | 0.13 | -41.54 | 322.53 |
| Mother’s perception of healthy body size for her child |  |  |  |  |
| Normal weight (vs. underweight) | -260.30 | 0.10 | -574.50 | 53.90 |
| Overweight (vs. underweight) | **-318.42** | **0.04** | **-627.94** | **-8.91** |
| Obese (vs. underweight) | **-314.02** | **0.05** | **-623.04** | **-5.01** |
| Total number of household assets | -4.79 | 0.71 | -29.66 | 20.08 |
| Female autonomy | 10.60 | 0.26 | -7.96 | 29.15 |
| Total amount spent on food for household | -3.56 | 0.75 | -25.84 | 18.72 |
| Total amount spent on special foods for children <5 years | -32.17 | 0.09 | -69.23 | 4.89 |

| **Table 26.** Predictors of legumes intake among mothers in the rainy season. Model estimates from multivariate linear regression. P<0.05 are bold. | | | | |
| --- | --- | --- | --- | --- |
| **Variable** | **Coefficient** | **P-value** | **Lower 95% CI** | **Upper 95% CI** |
| No child fever in last 2 weeks (vs. yes) | 29.94 | 0.10 | -5.61 | 65.49 |
| Total number of household assets | 5.89 | 0.63 | -17.84 | 29.63 |
| HFIAS score | **-24.80** | **0.02** | **-45.56** | **-4.04** |
| Female autonomy | 7.36 | 0.41 | -10.33 | 25.06 |
| Total amount spent on food for household | 4.15 | 0.71 | -17.59 | 25.89 |
| Total amount spent on special foods for children <5 years | 15.00 | 0.15 | -5.50 | 35.49 |
| Taste preference: legumes | **20.89** | **0.02** | **2.91** | **38.88** |

| **Table 27.** Predictors of legumes intake among children in the dry season. Model estimates from multivariate linear regression. P<0.05 are bold. | | | | |
| --- | --- | --- | --- | --- |
| **Variable** | **Coefficient** | **P-value** | **Lower 95% CI** | **Upper 95% CI** |
| Rural (vs. urban) | -31.32 | 0.14 | -73.29 | 10.66 |
| Main source of drinking water |  |  |  |  |
| Well (vs. piped) | 4.72 | 0.85 | -44.48 | 53.92 |
| Borehole (vs. piped) | 34.56 | 0.13 | -10.24 | 79.37 |
| Toilet facility used by household |  |  |  |  |
| Ventilated pit latrine (vs. flush toilet) | 0.00 | 1.00 | -61.31 | 61.31 |
| Pit latrine with roof (vs. flush toilet) | -2.07 | 0.95 | -60.82 | 56.68 |
| Traditional pit latrine (vs. flush toilet) | -14.61 | 0.67 | -82.60 | 53.37 |
| Purchase special foods for children  (vs. no) | -7.61 | 0.78 | -61.15 | 45.93 |
| No child fever in last 2 weeks (vs. yes) | 9.67 | 0.46 | -16.31 | 35.65 |
| Age of child | 8.56 | 0.19 | -4.16 | 21.28 |
| Total number of household assets | 13.74 | 0.12 | -3.71 | 31.20 |
| Female autonomy | 3.10 | 0.63 | -9.68 | 15.88 |
| Total amount spent on food for household | 9.56 | 0.22 | -5.64 | 24.77 |
| Total amount spent on special foods for children <5 years | -12.83 | 0.32 | -38.09 | 12.43 |
| Taste preference: legumes | **26.45** | **0.00** | **13.52** | **39.38** |

| **Table 28.** Predictors of legumes intake among children in the rainy season. Model estimates from multivariate linear regression. P<0.05 are bold. | | | | |
| --- | --- | --- | --- | --- |
| **Variable** | **Coefficient** | **P-value** | **Lower 95% CI** | **Upper 95% CI** |
| Dyad Type |  |  |  |  |
| Overweight mother, normal weight child (vs. both overweight) | 21.92 | 0.18 | -9.88 | 53.72 |
| Overweight child, normal weight mother (vs. both overweight) | 21.69 | 0.21 | -12.40 | 55.77 |
| Toilet facility used by household |  |  |  |  |
| Ventilated pit latrine (vs. flush toilet) | -83.84 | 0.05 | -168.51 | 0.83 |
| Pit latrine with roof (vs. flush toilet) | -72.34 | 0.09 | -156.25 | 11.58 |
| Traditional pit latrine (vs. flush toilet) | -67.61 | 0.16 | -162.65 | 27.44 |
| Purchase special foods for children  (vs. no) | 17.50 | 0.26 | -13.11 | 48.12 |
| No child fever in last 2 weeks (vs. yes) | 7.70 | 0.56 | -18.56 | 33.95 |
| No mother fever in last 2 weeks (vs. yes) | 21.64 | 0.16 | -8.87 | 52.14 |
| Total number of household assets | 3.82 | 0.68 | -14.34 | 21.98 |
| HFIAS score | -13.18 | 0.11 | -29.23 | 2.87 |
| Total amount spent on food for household | 9.33 | 0.25 | -6.75 | 25.40 |
| Total amount spent on special foods for children <5 years | -1.26 | 0.89 | -18.76 | 16.25 |
| Taste preference: legumes | **22.86** | **0.00** | **9.21** | **36.50** |

| **Table 29.** Predictors of vegetable intake among mothers in the dry season. Model estimates from multivariate linear regression. P<0.05 are bold. | | | | |
| --- | --- | --- | --- | --- |
| **Variable** | **Coefficient** | **P-value** | **Lower 95% CI** | **Upper 95% CI** |
| Rural (vs. urban) | 4.53 | 0.92 | -80.31 | 89.37 |
| Main source of drinking water |  |  |  |  |
| Well (vs. piped) | -84.03 | 0.10 | -184.95 | 16.89 |
| Borehole (vs. piped) | 15.47 | 0.74 | -75.62 | 106.57 |
| Who purchases most food the family consumes |  |  |  |  |
| Husband/partner (vs. mother) | -13.08 | 0.72 | -83.68 | 57.52 |
| Both (vs. mother) | -50.79 | 0.28 | -142.98 | 41.41 |
| Other family member (vs. mother) | 62.39 | 0.24 | -42.67 | 167.45 |
| Purchase special foods for children  (vs. no) | 6.69 | 0.81 | -48.37 | 61.75 |
| Total number of household assets | 15.53 | 0.36 | -17.53 | 48.58 |
| HFIAS score | -13.45 | 0.35 | -41.83 | 14.94 |
| Taste preference: vegetables | **32.26** | **0.02** | **6.22** | **58.30** |

| **Table 30.** Predictors of vegetable intake among mothers in the rainy season. Model estimates from multivariate linear regression. P<0.05 are bold. | | | | |
| --- | --- | --- | --- | --- |
| **Variable** | **Coefficient** | **P-value** | **Lower 95% CI** | **Upper 95% CI** |
| Dyad Type |  |  |  |  |
| Overweight mother, normal weight child (vs. both overweight) | **-147.22** | **0.00** | **-232.74** | **-61.70** |
| Overweight child, normal weight mother (vs. both overweight) | 4.22 | 0.93 | -88.62 | 97.05 |
| Rural (vs. urban) | -5.52 | 0.93 | -129.81 | 118.76 |
| Main source of drinking water |  |  |  |  |
| Well (vs. piped) | 41.57 | 0.60 | -115.46 | 198.60 |
| Borehole (vs. piped) | **129.57** | **0.05** | **0.80** | **258.33** |
| Who purchases most food the family consumes |  |  |  |  |
| Husband/partner (vs. mother) | -23.88 | 0.64 | -123.91 | 76.14 |
| Both (vs. mother) | -16.91 | 0.79 | -141.87 | 108.04 |
| Other family member (vs. mother) | -106.55 | 0.16 | -255.19 | 42.09 |
| No mother cough in last 2 weeks (vs. yes) | 62.34 | 0.13 | -18.74 | 143.42 |
| Number of children <5 years in household | -19.96 | 0.26 | -54.98 | 15.06 |
| Total amount spent on food for household | 0.13 | 1.00 | -39.50 | 39.76 |
| Taste preference: vegetables | **53.78** | **0.00** | **17.07** | **90.49** |

| **Table 31.** Predictors of vegetable intake among children in the dry season. Model estimates from multivariate linear regression. P<0.05 are bold. | | | | |
| --- | --- | --- | --- | --- |
| **Variable** | **Coefficient** | **P-value** | **Lower 95% CI** | **Upper 95% CI** |
| Kasungu district (vs. Lilongwe) | 13.96 | 0.20 | -7.38 | 35.30 |
| Toilet facility used by household |  |  |  |  |
| Ventilated pit latrine (vs. flush toilet) | 35.81 | 0.17 | -14.93 | 86.54 |
| Pit latrine with roof (vs. flush toilet) | 21.96 | 0.35 | -24.36 | 68.27 |
| Traditional pit latrine (vs. flush toilet) | 1.49 | 0.96 | -50.88 | 53.86 |
| Age of child | **25.95** | **0.00** | **15.32** | **36.58** |
| How long it takes to get to nearest market/shop to purchase food | 1.99 | 0.71 | -8.64 | 12.63 |
| Total amount spent on special foods for children <5 years | **-11.72** | **0.03** | **-22.51** | **-0.93** |
| Taste preference: vegetables | **17.88** | **0.00** | **7.01** | **28.74** |

| **Table 32.** Predictors of vegetable intake among children in the rainy season. Model estimates from multivariate linear regression. P<0.05 are bold. | | | | |
| --- | --- | --- | --- | --- |
| **Variable** | **Coefficient** | **P-value** | **Lower 95% CI** | **Upper 95% CI** |
| Dyad Type |  |  |  |  |
| Overweight mother, normal weight child (vs. both overweight) | **-109.86** | **0.00** | **-155.30** | **-64.42** |
| Overweight child, normal weight mother (vs. both overweight) | -35.19 | 0.15 | -83.45 | 13.06 |
| Rural (vs. urban) | 3.08 | 0.93 | -61.49 | 67.65 |
| Mother secondary education or higher (vs. less) | -7.10 | 0.74 | -49.84 | 35.64 |
| Main source of drinking water |  |  |  |  |
| Well (vs. piped) | 8.83 | 0.83 | -74.21 | 91.87 |
| Borehole (vs. piped) | **69.65** | **0.05** | **1.65** | **137.65** |
| Who purchases most food the family consumes |  |  |  |  |
| Husband/partner (vs. mother) | -48.00 | 0.08 | -101.98 | 5.98 |
| Both (vs. mother) | -50.26 | 0.13 | -115.30 | 14.79 |
| Other family member (vs. mother) | **-97.42** | **0.02** | **-179.15** | **-15.68** |
| No mother fever in last 2 weeks (vs. yes) | 1.99 | 0.93 | -42.56 | 46.54 |
| Age of child | 40.65 | 0.00 | 20.69 | 60.60 |
| Total number of household assets | -7.44 | 0.58 | -33.68 | 18.81 |
| HFIAS score | -17.68 | 0.11 | -39.16 | 3.80 |
| Female autonomy | **-25.83** | **0.01** | **-45.85** | **-5.81** |
| Total amount spent on food for household | -2.90 | 0.80 | -25.20 | 19.41 |
| How long it takes to get to nearest market/shop to purchase food | -11.61 | 0.22 | -30.16 | 6.94 |
| Taste preference: vegetables | **23.76** | **0.02** | **4.68** | **42.83** |

| **Table 33.** Predictors of fruit intake among mothers in the dry season. Model estimates from multivariate linear regression. P<0.05 are bold. | | | | |
| --- | --- | --- | --- | --- |
| **Variable** | **Coefficient** | **P-value** | **Lower 95% CI** | **Upper 95% CI** |
| Rural (vs. urban) | 34.20 | 0.29 | -28.61 | 97.01 |
| Mother secondary education or higher (vs. less) | -5.42 | 0.81 | -48.90 | 38.06 |
| Main source of drinking water |  |  |  |  |
| Well (vs. piped) | -18.10 | 0.63 | -90.88 | 54.68 |
| Borehole (vs. piped) | -0.51 | 0.99 | -67.04 | 66.02 |
| Toilet facility used by household |  |  |  |  |
| Ventilated pit latrine (vs. flush toilet) | 45.96 | 0.32 | -45.67 | 137.60 |
| Pit latrine with roof (vs. flush toilet) | 56.78 | 0.21 | -31.60 | 145.17 |
| Traditional pit latrine (vs. flush toilet) | 31.56 | 0.55 | -72.02 | 135.14 |
| Who purchases most food the family consumes |  |  |  |  |
| Husband/partner (vs. mother) | -9.07 | 0.74 | -61.89 | 43.75 |
| Both (vs. mother) | -26.55 | 0.44 | -94.62 | 41.52 |
| Other family member (vs. mother) | -4.01 | 0.92 | -81.19 | 73.16 |
| Purchase special foods for children  (vs. no) | 19.58 | 0.34 | -20.94 | 60.10 |
| Mother’s body size preference for herself |  |  |  |  |
| Normal weight (vs. underweight) | 28.62 | 0.75 | -150.75 | 207.99 |
| Overweight (vs. underweight) | 6.79 | 0.94 | -170.85 | 184.43 |
| Obese (vs. underweight) | 70.31 | 0.45 | -112.82 | 253.44 |
| Mother’s perception of healthy body size for herself |  |  |  |  |
| Overweight (vs. normal weight) | -23.04 | 0.60 | -110.13 | 64.04 |
| Obese (vs. normal weight) | -69.47 | 0.13 | -159.63 | 20.70 |
| Total number of household assets | 15.79 | 0.26 | -11.64 | 43.21 |
| HFIAS score | **-22.37** | **0.04** | **-43.73** | **-1.02** |
| Total amount spent on food for household | -2.64 | 0.82 | -25.02 | 19.74 |
| How long it takes to get to nearest market/shop to purchase food | -11.53 | 0.25 | -31.10 | 8.03 |

| **Table 34.** Predictors of fruit intake among mothers in the rainy season. Model estimates from multivariate linear regression. P<0.05 are bold. | | | | |
| --- | --- | --- | --- | --- |
| **Variable** | **Coefficient** | **P-value** | **Lower 95% CI** | **Upper 95% CI** |
| Dyad Type |  |  |  |  |
| Overweight mother, normal weight child (vs. both overweight) | **70.77** | **0.01** | **16.49** | **125.06** |
| Overweight child, normal weight mother (vs. both overweight) | 1.54 | 0.96 | -55.65 | 58.73 |
| Kasungu district (vs. Lilongwe) | **-45.41** | **0.04** | **-89.30** | **-1.52** |
| Rural (vs. urban) | 72.45 | 0.06 | -4.28 | 149.18 |
| Mother secondary education or higher (vs. less) | -15.27 | 0.55 | -65.63 | 35.08 |
| Main source of drinking water |  |  |  |  |
| Well (vs. piped) | -96.64 | 0.06 | -197.87 | 4.60 |
| Borehole (vs. piped) | **-88.74** | **0.03** | **-170.61** | **-6.87** |
| Toilet facility used by household |  |  |  |  |
| Ventilated pit latrine (vs. flush toilet) | 4.17 | 0.96 | -139.97 | 148.31 |
| Pit latrine with roof (vs. flush toilet) | 10.91 | 0.88 | -133.97 | 155.79 |
| Traditional pit latrine (vs. flush toilet) | 37.03 | 0.66 | -126.94 | 200.99 |
| Mother’s perception of healthy body size for herself |  |  |  |  |
| Overweight (vs. normal weight) | 26.74 | 0.62 | -78.84 | 132.32 |
| Obese (vs. normal weight) | 4.98 | 0.93 | -100.82 | 110.77 |
| Total number of household assets | 33.03 | 0.05 | -0.36 | 66.43 |
| HFIAS score | -4.88 | 0.71 | -30.87 | 21.11 |
| Total amount spent on food for household | -20.41 | 0.16 | -48.75 | 7.94 |
| Total amount spent on special foods for children <5 years | 17.09 | 0.20 | -8.86 | 43.05 |

| **Table 35.** Predictors of fruit intake among children in the dry season. Model estimates from multivariate linear regression. P<0.05 are bold. | | | | |
| --- | --- | --- | --- | --- |
| **Variable** | **Coefficient** | **P-value** | **Lower 95% CI** | **Upper 95% CI** |
| Rural (vs. urban) | -3.55 | 0.81 | -32.98 | 25.88 |
| Mother secondary education or higher (vs. less) | 5.55 | 0.59 | -14.67 | 25.76 |
| Main source of drinking water |  |  |  |  |
| Well (vs. piped) | 10.34 | 0.55 | -23.64 | 44.32 |
| Borehole (vs. piped) | 15.79 | 0.32 | -15.35 | 46.92 |
| Toilet facility used by household |  |  |  |  |
| Ventilated pit latrine (vs. flush toilet) | 12.88 | 0.55 | -29.51 | 55.27 |
| Pit latrine with roof (vs. flush toilet) | 14.51 | 0.48 | -26.08 | 55.10 |
| Traditional pit latrine (vs. flush toilet) | 0.30 | 0.99 | -46.67 | 47.26 |
| Purchase special foods for children  (vs. no) | 6.45 | 0.50 | -12.19 | 25.10 |
| Mother’s body size preference for herself |  |  |  |  |
| Normal weight (vs. underweight) | 22.86 | 0.59 | -61.07 | 106.78 |
| Overweight (vs. underweight) | 16.33 | 0.70 | -66.61 | 99.27 |
| Obese (vs. underweight) | 53.60 | 0.22 | -32.06 | 139.27 |
| Mother’s perception of healthy body size for herself |  |  |  |  |
| Overweight (vs. normal weight) | 8.07 | 0.71 | -34.26 | 50.39 |
| Obese (vs. normal weight) | 3.41 | 0.89 | -43.58 | 50.40 |
| Mother’s perception of healthy body size for her child |  |  |  |  |
| Normal weight (vs. underweight) | -20.61 | 0.79 | -169.85 | 128.63 |
| Overweight (vs. underweight) | 1.42 | 0.99 | -144.99 | 147.82 |
| Obese (vs. underweight) | -22.11 | 0.77 | -169.25 | 125.04 |
| No child fever in last 2 weeks (vs. yes) | 4.62 | 0.61 | -13.32 | 22.57 |
| Age of child | 4.69 | 0.30 | -4.24 | 13.62 |
| Total number of household assets | 8.58 | 0.19 | -4.32 | 21.48 |
| HFIAS score | -5.94 | 0.24 | -15.94 | 4.06 |
| Total amount spent on food for household | -2.57 | 0.63 | -12.98 | 7.84 |

| **Table 36.** Predictors of fruit intake among children in the rainy season. Model estimates from multivariate linear regression. P<0.05 are bold. | | | | |
| --- | --- | --- | --- | --- |
| **Variable** | **Coefficient** | **P-value** | **Lower 95% CI** | **Upper 95% CI** |
| Dyad Type |  |  |  |  |
| Overweight mother, normal weight child (vs. both overweight) | 24.39 | 0.05 | -0.22 | 48.99 |
| Overweight child, normal weight mother (vs. both overweight) | -2.49 | 0.85 | -29.17 | 24.18 |
| Rural (vs. urban) | 9.19 | 0.60 | -24.99 | 43.36 |
| Mother secondary education or higher (vs. less) | -3.31 | 0.77 | -25.84 | 19.22 |
| Main source of drinking water |  |  |  |  |
| Well (vs. piped) | 6.70 | 0.77 | -38.74 | 52.15 |
| Borehole (vs. piped) | -10.62 | 0.57 | -47.47 | 26.24 |
| Toilet facility used by household |  |  |  |  |
| Ventilated pit latrine (vs. flush toilet) | -2.60 | 0.94 | -66.32 | 61.12 |
| Pit latrine with roof (vs. flush toilet) | -15.98 | 0.62 | -80.01 | 48.05 |
| Traditional pit latrine (vs. flush toilet) | -9.26 | 0.80 | -82.59 | 64.07 |
| Mother’s perception of healthy body size for herself |  |  |  |  |
| Overweight (vs. normal weight) | -1.12 | 0.96 | -48.64 | 46.41 |
| Obese (vs. normal weight) | 7.84 | 0.74 | -39.18 | 54.86 |
| No child diarrhea in last 2 weeks (vs. yes) | 6.78 | 0.55 | -15.49 | 29.06 |
| Age of mother | 5.19 | 0.34 | -5.59 | 15.97 |
| Age of child | 10.17 | 0.07 | -0.84 | 21.19 |
| Total number of household assets | **19.38** | **0.01** | **4.35** | **34.40** |
| HFIAS score | -3.93 | 0.51 | -15.63 | 7.77 |
| Total amount spent on food for household | -10.72 | 0.10 | -23.41 | 1.98 |
| Total amount spent on special foods for children <5 years | 10.63 | 0.07 | -0.91 | 22.17 |

| **Table 37.** Predictors of meat and egg intake among mothers in the dry season. Model estimates from multivariate linear regression. P<0.05 are bold. | | | | |  |
| --- | --- | --- | --- | --- | --- |
| **Variable** | **Coefficient** | **P-value** | **Lower 95% CI** | **Upper 95% CI** | |
| Rural (vs. urban) | 4.29 | 0.65 | -14.16 | 22.75 | |
| Mother secondary education or higher (vs. less) | -3.32 | 0.61 | -16.15 | 9.51 | |
| Main source of drinking water |  |  |  |  | |
| Well (vs. piped) | -6.04 | 0.58 | -27.60 | 15.53 | |
| Borehole (vs. piped) | -3.55 | 0.72 | -23.31 | 16.21 | |
| Toilet facility used by household |  |  |  |  | |
| Ventilated pit latrine (vs. flush toilet) | 11.20 | 0.42 | -15.91 | 38.30 | |
| Pit latrine with roof (vs. flush toilet) | 22.91 | 0.09 | -3.39 | 49.20 | |
| Traditional pit latrine (vs. flush toilet) | 20.37 | 0.19 | -10.37 | 51.11 | |
| Who purchases most food the family consumes |  |  |  |  | |
| Husband/partner (vs. mother) | -1.98 | 0.81 | -17.71 | 13.76 | |
| Both (vs. mother) | 9.74 | 0.34 | -10.30 | 29.79 | |
| Other family member (vs. mother) | **-27.01** | **0.02** | **-49.90** | **-4.12** | |
| Other transport to buy food (vs. walk) | **44.72** | **0.00** | **19.24** | **70.20** | |
| Purchase special foods for children  (vs. no) | 8.52 | 0.16 | -3.34 | 20.37 | |
| Mother’s perception of healthy body size for her child |  |  |  |  | |
| Normal weight (vs. underweight) | -22.10 | 0.64 | -114.91 | 70.72 | |
| Overweight (vs. underweight) | -5.75 | 0.90 | -97.12 | 85.62 | |
| Obese (vs. underweight) | -13.76 | 0.77 | -104.90 | 77.38 | |
| Total number of household assets | **18.51** | **0.00** | **10.28** | **26.73** | |
| HFIAS score | -5.78 | 0.07 | -12.01 | 0.46 | |
| Total amount spent on food for household | 0.69 | 0.84 | -5.90 | 7.27 | |
| How long it takes to get to nearest market/shop to purchase food | 1.98 | 0.51 | -3.98 | 7.93 | |

| **Table 38.** Predictors of meat and egg intake among mothers in the rainy season. Model estimates from multivariate linear regression. P<0.05 are bold. | | | | |
| --- | --- | --- | --- | --- |
| **Variable** | **Coefficient** | **P-value** | **Lower 95% CI** | **Upper 95% CI** |
| Female sex of child (vs. male) | **16.66** | **0.01** | **4.72** | **28.60** |
| Rural (vs. urban) | 3.27 | 0.75 | -17.28 | 23.82 |
| Mother secondary education or higher (vs. less) | 3.82 | 0.58 | -9.68 | 17.32 |
| Main source of drinking water |  |  |  |  |
| Well (vs. piped) | -13.03 | 0.34 | -39.80 | 13.73 |
| Borehole (vs. piped) | -2.21 | 0.84 | -23.78 | 19.36 |
| Toilet facility used by household |  |  |  |  |
| Ventilated pit latrine (vs. flush toilet) | 15.99 | 0.43 | -23.59 | 55.57 |
| Pit latrine with roof (vs. flush toilet) | 19.50 | 0.33 | -20.02 | 59.03 |
| Traditional pit latrine (vs. flush toilet) | 26.58 | 0.24 | -18.16 | 71.33 |
| Purchase special foods for children  (vs. no) | 2.55 | 0.73 | -12.05 | 17.16 |
| Mother’s body size preference for herself |  |  |  |  |
| Normal weight (vs. underweight) | -9.76 | 0.78 | -79.36 | 59.84 |
| Overweight (vs. underweight) | -4.47 | 0.90 | -73.69 | 64.75 |
| Obese (vs. underweight) | -6.09 | 0.87 | -76.44 | 64.26 |
| Mother’s perception of healthy body size for herself |  |  |  |  |
| Overweight (vs. normal weight) | -12.72 | 0.38 | -41.45 | 16.01 |
| Obese (vs. normal weight) | -11.15 | 0.46 | -40.68 | 18.38 |
| No child fever in last 2 weeks (vs. yes) | 1.83 | 0.77 | -10.56 | 14.21 |
| No mother fever in last 2 weeks (vs. yes) | 7.46 | 0.31 | -6.87 | 21.78 |
| No mother cough in last 2 weeks (vs. yes) | 7.77 | 0.27 | -5.96 | 21.50 |
| Total number of household assets | 5.70 | 0.22 | -3.40 | 14.80 |
| HFIAS score | -6.41 | 0.08 | -13.69 | 0.86 |
| Total amount spent on food for household | 7.31 | 0.06 | -0.30 | 14.91 |
| Total amount spent on special foods for children <5 years | **13.26** | **0.00** | **5.00** | **21.51** |
| Taste preference: meat and eggs | -3.09 | 0.34 | -9.43 | 3.26 |

| **Table 39.** Predictors of meat and egg intake among children in the dry season. Model estimates from multivariate linear regression. P<0.05 are bold. | | | | |
| --- | --- | --- | --- | --- |
| **Variable** | **Coefficient** | **P-value** | **Lower 95% CI** | **Upper 95% CI** |
| Rural (vs. urban) | -11.21 | 0.12 | -25.36 | 2.95 |
| Mother secondary education or higher (vs. less) | 3.11 | 0.53 | -6.72 | 12.94 |
| Main source of drinking water |  |  |  |  |
| Well (vs. piped) | 1.28 | 0.88 | -15.42 | 17.99 |
| Borehole (vs. piped) | 13.52 | 0.08 | -1.61 | 28.66 |
| Purchase special foods for children  (vs. no) | -0.04 | 0.99 | -9.04 | 8.95 |
| No child diarrhea in last 2 weeks (vs. yes) | 1.27 | 0.80 | -8.64 | 11.17 |
| No child cough in last 2 weeks (vs. yes) | 7.80 | 0.09 | -1.20 | 16.79 |
| Age of mother | -0.22 | 0.92 | -4.66 | 4.23 |
| Age of child | **6.80** | **0.00** | **2.42** | **11.18** |
| Total number of household assets | **6.40** | **0.03** | **0.50** | **12.30** |
| HFIAS score | 1.79 | 0.46 | -3.00 | 6.57 |
| Total amount spent on food for household | 1.55 | 0.56 | -3.61 | 6.71 |

| **Table 40.** Predictors of meat and egg intake among children in the rainy season. Model estimates from multivariate linear regression. P<0.05 are bold. | | | | |
| --- | --- | --- | --- | --- |
| **Variable** | **Coefficient** | **P-value** | **Lower 95% CI** | **Upper 95% CI** |
| Female sex of child (vs. male) | **10.98** | **0.01** | **3.21** | **18.76** |
| Rural (vs. urban) | -4.02 | 0.56 | -17.54 | 9.51 |
| Mother secondary education or higher (vs. less) | 7.66 | 0.10 | -1.34 | 16.66 |
| Main source of drinking water |  |  |  |  |
| Well (vs. piped) | -3.42 | 0.70 | -20.79 | 13.95 |
| Borehole (vs. piped) | 9.37 | 0.20 | -4.84 | 23.59 |
| Toilet facility used by household |  |  |  |  |
| Ventilated pit latrine (vs. flush toilet) | -3.62 | 0.78 | -28.99 | 21.74 |
| Pit latrine with roof (vs. flush toilet) | 9.32 | 0.47 | -16.01 | 34.66 |
| Traditional pit latrine (vs. flush toilet) | 14.23 | 0.33 | -14.38 | 42.85 |
| Who purchases most food the family consumes |  |  |  |  |
| Husband/partner (vs. mother) | -6.51 | 0.26 | -17.85 | 4.82 |
| Both (vs. mother) | 11.85 | 0.10 | -2.10 | 25.80 |
| Other family member (vs. mother) | 0.67 | 0.94 | -15.94 | 17.28 |
| Purchase special foods for children  (vs. no) | 1.90 | 0.69 | -7.55 | 11.34 |
| No child fever in last 2 weeks (vs. yes) | 4.56 | 0.26 | -3.44 | 12.56 |
| No mother fever in last 2 weeks (vs. yes) | 4.45 | 0.36 | -5.01 | 13.92 |
| No mother cough in last 2 weeks (vs. yes) | **9.83** | **0.03** | **0.82** | **18.83** |
| Total number of household assets | 2.20 | 0.46 | -3.65 | 8.05 |
| HFIAS score | **-5.97** | **0.01** | **-10.72** | **-1.22** |
| Total amount spent on food for household | **6.37** | **0.01** | **1.41** | **11.34** |
| Total amount spent on special foods for children <5 years | **6.84** | **0.01** | **1.53** | **12.15** |
| Taste preference: meat and eggs | -2.18 | 0.29 | -6.26 | 1.89 |

| **Table 41.** Predictors of fish intake among mothers in the dry season. Model estimates from multivariate linear regression. P<0.05 are bold. | | | | |
| --- | --- | --- | --- | --- |
| **Variable** | **Coefficient** | **P-value** | **Lower 95% CI** | **Upper 95% CI** |
| Rural (vs. urban) | -12.86 | 0.16 | -30.61 | 4.89 |
| Main source of drinking water |  |  |  |  |
| Well (vs. piped) | -4.47 | 0.67 | -25.21 | 16.27 |
| Borehole (vs. piped) | 0.49 | 0.96 | -18.46 | 19.45 |
| Mother’s perception of healthy body size for herself |  |  |  |  |
| Overweight (vs. normal weight) | -8.22 | 0.51 | -32.60 | 16.17 |
| Obese (vs. normal weight) | -9.43 | 0.45 | -34.05 | 15.19 |
| No child fever in last 2 weeks (vs. yes) | **11.81** | **0.03** | **0.99** | **22.62** |
| Total number of household assets | 1.98 | 0.58 | -5.00 | 8.96 |
| Total amount spent on food for household | -1.78 | 0.58 | -8.10 | 4.53 |

| **Table 42.** Predictors of fish intake among mothers in the rainy season. Model estimates from multivariate linear regression. P<0.05 are bold. | | | | |
| --- | --- | --- | --- | --- |
| **Variable** | **Coefficient** | **P-value** | **Lower 95% CI** | **Upper 95% CI** |
| Rural (vs. urban) | -16.02 | 0.36 | -50.71 | 18.67 |
| Main source of drinking water |  |  |  |  |
| Well (vs. piped) | -25.70 | 0.26 | -70.65 | 19.26 |
| Borehole (vs. piped) | -9.60 | 0.61 | -46.22 | 27.02 |
| No child fever in last 2 weeks (vs. yes) | 1.80 | 0.87 | -19.27 | 22.88 |
| No child cough in last 2 weeks (vs. yes) | 1.56 | 0.88 | -19.16 | 22.28 |
| No mother fever in last 2 weeks (vs. yes) | 16.02 | 0.19 | -7.90 | 39.94 |
| Total number of household assets | -1.13 | 0.88 | -15.24 | 12.98 |
| HFIAS score | -2.26 | 0.70 | -13.64 | 9.11 |
| Total amount spent on food for household | -4.65 | 0.46 | -17.06 | 7.76 |
| How long it takes to get to nearest market/shop to purchase food | **-10.27** | **0.04** | **-20.23** | **-0.31** |
| Total amount spent on special foods for children <5 years | 3.23 | 0.58 | -8.31 | 14.78 |

| **Table 43.** Predictors of fish intake among children in the dry season. Model estimates from multivariate linear regression. P<0.05 are bold. | | | | |
| --- | --- | --- | --- | --- |
| **Variable** | **Coefficient** | **P-value** | **Lower 95% CI** | **Upper 95% CI** |
| Rural (vs. urban) | **-6.11** | **0.02** | **-11.26** | **-0.97** |
| Toilet facility used by household |  |  |  |  |
| Ventilated pit latrine (vs. flush toilet) | 3.73 | 0.51 | -7.35 | 14.81 |
| Pit latrine with roof (vs. flush toilet) | 4.63 | 0.37 | -5.60 | 14.86 |
| Traditional pit latrine (vs. flush toilet) | 0.86 | 0.89 | -10.97 | 12.70 |
| Purchase special foods for children  (vs. no) | 0.51 | 0.92 | -8.83 | 9.85 |
| Age of child | **6.20** | **0.00** | **3.83** | **8.57** |
| Total amount spent on special foods for children <5 years | -1.65 | 0.48 | -6.19 | 2.90 |
| Taste preference: fish | **3.54** | **0.00** | **1.13** | **5.94** |

| **Table 44.** Predictors of fish intake among children in the rainy season. Model estimates from multivariate linear regression. P<0.05 are bold. | | | | |
| --- | --- | --- | --- | --- |
| **Variable** | **Coefficient** | **P-value** | **Lower 95% CI** | **Upper 95% CI** |
| Kasungu district (vs. Lilongwe) | 5.21 | 0.33 | -5.33 | 15.74 |
| Rural (vs. urban) | -4.63 | 0.62 | -23.24 | 13.97 |
| Main source of drinking water |  |  |  |  |
| Well (vs. piped) | -19.53 | 0.11 | -43.57 | 4.50 |
| Borehole (vs. piped) | -8.51 | 0.40 | -28.38 | 11.36 |
| Who purchases most food the family consumes |  |  |  |  |
| Husband/partner (vs. mother) | 1.24 | 0.87 | -13.77 | 16.25 |
| Both (vs. mother) | -12.13 | 0.20 | -30.72 | 6.47 |
| Other family member (vs. mother) | -16.12 | 0.15 | -38.31 | 6.06 |
| No child fever in last 2 weeks (vs. yes) | -1.85 | 0.75 | -13.26 | 9.56 |
| No child cough in last 2 weeks (vs. yes) | 2.65 | 0.64 | -8.62 | 13.92 |
| No mother fever in last 2 weeks (vs. yes) | 9.97 | 0.13 | -2.90 | 22.83 |
| Age of child | 5.18 | 0.06 | -0.17 | 10.53 |
| Total number of household assets | 1.31 | 0.73 | -6.03 | 8.65 |
| HFIAS score | 0.31 | 0.92 | -5.74 | 6.36 |
| Total amount spent on food for household | -1.60 | 0.62 | -8.00 | 4.80 |
| How long it takes to get to nearest market/shop to purchase food | -5.12 | 0.06 | -10.44 | 0.21 |

| **Table 45.** Predictors of dairy intake among mothers in the dry season. Model estimates from multivariate linear regression. P<0.05 are bold. | | | | |
| --- | --- | --- | --- | --- |
| **Variable** | **Coefficient** | **P-value** | **Lower 95% CI** | **Upper 95% CI** |
| Rural (vs. urban) | -16.55 | 0.10 | -36.14 | 3.04 |
| Mother secondary education or higher (vs. less) | 10.42 | 0.13 | -2.90 | 23.73 |
| Main source of drinking water |  |  |  |  |
| Well (vs. piped) | -0.71 | 0.95 | -23.51 | 22.09 |
| Borehole (vs. piped) | 11.32 | 0.29 | -9.47 | 32.12 |
| Toilet facility used by household |  |  |  |  |
| Ventilated pit latrine (vs. flush toilet) | -4.92 | 0.73 | -33.25 | 23.41 |
| Pit latrine with roof (vs. flush toilet) | -2.20 | 0.87 | -29.41 | 25.01 |
| Traditional pit latrine (vs. flush toilet) | 6.55 | 0.68 | -24.74 | 37.85 |
| Other transport to buy food (vs. walk) | 16.98 | 0.21 | -9.56 | 43.53 |
| Purchase special foods for children  (vs. no) | **55.40** | **0.00** | **30.88** | **79.92** |
| Mother’s perception of healthy body size for her child |  |  |  |  |
| Normal weight (vs. underweight) | **-273.57** | **0.00** | **-371.23** | **-175.90** |
| Overweight (vs. underweight) | **-295.53** | **0.00** | **-391.77** | **-199.30** |
| Obese (vs. underweight) | **-285.46** | **0.00** | **-381.59** | **-189.33** |
| No mother cough in last 2 weeks (vs. yes) | -7.38 | 0.26 | -20.31 | 5.54 |
| Total number of household assets | 6.49 | 0.14 | -2.20 | 15.17 |
| HFIAS score | 1.26 | 0.70 | -5.26 | 7.77 |
| Female autonomy | 4.11 | 0.17 | -1.76 | 9.97 |
| Total amount spent on food for household | -0.52 | 0.89 | -7.67 | 6.63 |
| Total amount spent on special foods for children <5 years | **23.77** | **0.00** | **12.17** | **35.37** |
| Taste preference: dairy | -0.16 | 0.96 | -6.20 | 5.87 |

| **Table 46.** Predictors of dairy intake among mothers in the rainy season. Model estimates from multivariate linear regression. P<0.05 are bold. | | | | |
| --- | --- | --- | --- | --- |
| **Variable** | **Coefficient** | **P-value** | **Lower 95% CI** | **Upper 95% CI** |
| Dyad Type |  |  |  |  |
| Overweight mother, normal weight child (vs. both overweight) | 3.77 | 0.68 | -14.25 | 21.79 |
| Overweight child, normal weight mother (vs. both overweight) | 2.24 | 0.81 | -16.10 | 20.58 |
| Rural (vs. urban) | 4.80 | 0.70 | -19.53 | 29.12 |
| Mother secondary education or higher (vs. less) | 5.83 | 0.48 | -10.34 | 21.99 |
| Main source of drinking water |  |  |  |  |
| Well (vs. piped) | 0.86 | 0.96 | -31.11 | 32.84 |
| Borehole (vs. piped) | 9.15 | 0.49 | -16.70 | 35.00 |
| Toilet facility used by household |  |  |  |  |
| Ventilated pit latrine (vs. flush toilet) | -5.77 | 0.81 | -52.11 | 40.57 |
| Pit latrine with roof (vs. flush toilet) | -13.47 | 0.57 | -59.59 | 32.66 |
| Traditional pit latrine (vs. flush toilet) | -12.50 | 0.64 | -64.42 | 39.41 |
| Purchase special foods for children  (vs. no) | 10.24 | 0.23 | -6.62 | 27.10 |
| Mother’s perception of healthy body size for herself |  |  |  |  |
| Overweight (vs. normal weight) | -7.16 | 0.68 | -40.93 | 26.62 |
| Obese (vs. normal weight) | 1.04 | 0.95 | -32.57 | 34.66 |
| No child fever in last 2 weeks (vs. yes) | 12.37 | 0.10 | -2.21 | 26.96 |
| No mother diarrhea in last 2 weeks (vs. yes) | 13.91 | 0.26 | -10.56 | 38.37 |
| Total number of household assets | 0.73 | 0.89 | -10.04 | 11.50 |
| HFIAS score | -2.69 | 0.55 | -11.52 | 6.14 |
| Female autonomy | -0.45 | 0.91 | -7.94 | 7.04 |
| Total amount spent on food for household | **18.18** | **0.00** | **9.10** | **27.26** |
| Total amount spent on special foods for children <5 years | 3.57 | 0.47 | -6.18 | 13.31 |
| Taste preference: dairy | 7.40 | 0.06 | -0.42 | 15.22 |

| **Table 47.** Predictors of dairy intake among children in the dry season. Model estimates from multivariate linear regression. P<0.05 are bold. | | | | |
| --- | --- | --- | --- | --- |
| **Variable** | **Coefficient** | **P-value** | **Lower 95% CI** | **Upper 95% CI** |
| Rural (vs. urban) | 0.85 | 0.97 | -39.79 | 41.49 |
| Mother secondary education or higher (vs. less) | 13.70 | 0.33 | -14.19 | 41.58 |
| Main source of drinking water |  |  |  |  |
| Well (vs. piped) | -24.12 | 0.31 | -70.41 | 22.18 |
| Borehole (vs. piped) | -5.46 | 0.80 | -48.16 | 37.23 |
| Toilet facility used by household |  |  |  |  |
| Ventilated pit latrine (vs. flush toilet) | -48.23 | 0.11 | -107.07 | 10.62 |
| Pit latrine with roof (vs. flush toilet) | -17.09 | 0.55 | -73.70 | 39.52 |
| Traditional pit latrine (vs. flush toilet) | -16.78 | 0.62 | -82.85 | 49.29 |
| Who purchases most food the family consumes |  |  |  |  |
| Husband/partner (vs. mother) | 4.98 | 0.78 | -30.10 | 40.05 |
| Both (vs. mother) | -1.71 | 0.94 | -44.98 | 41.55 |
| Other family member (vs. mother) | 14.56 | 0.61 | -41.21 | 70.32 |
| Other transport to buy food (vs. walk) | 12.73 | 0.64 | -40.99 | 66.45 |
| Purchase special foods for children  (vs. no) | 15.36 | 0.24 | -10.37 | 41.09 |
| Mother’s perception of healthy body size for herself |  |  |  |  |
| Overweight (vs. normal weight) | 46.84 | 0.11 | -10.02 | 103.69 |
| Obese (vs. normal weight) | **67.55** | **0.04** | **4.70** | **130.40** |
| Mother’s perception of healthy body size for her child |  |  |  |  |
| Normal weight (vs. underweight) | -160.86 | 0.12 | -360.90 | 39.18 |
| Overweight (vs. underweight) | **-262.76** | **0.01** | **-458.97** | **-66.55** |
| Obese (vs. underweight) | **-264.84** | **0.01** | **-461.56** | **-68.11** |
| No child diarrhea in last 2 weeks (vs. yes) | 7.66 | 0.58 | -19.50 | 34.83 |
| No mother fever in last 2 weeks (vs. yes) | 19.98 | 0.31 | -18.30 | 58.26 |
| Total number of household assets | 12.15 | 0.18 | -5.48 | 29.79 |
| HFIAS score | -2.73 | 0.69 | -16.19 | 10.73 |
| Female autonomy | 6.65 | 0.34 | -7.03 | 20.33 |
| Total amount spent on food for household | **27.31** | **0.00** | **13.06** | **41.56** |

| **Table 48.** Predictors of dairy intake among children in the rainy season. Model estimates from multivariate linear regression. P<0.05 are bold. | | | | |
| --- | --- | --- | --- | --- |
| **Variable** | **Coefficient** | **P-value** | **Lower 95% CI** | **Upper 95% CI** |
| Rural (vs. urban) | 0.41 | 0.98 | -30.84 | 31.66 |
| Mother secondary education or higher (vs. less) | 2.16 | 0.84 | -18.69 | 23.02 |
| Main source of drinking water |  |  |  |  |
| Well (vs. piped) | -0.42 | 0.98 | -41.26 | 40.43 |
| Borehole (vs. piped) | 4.96 | 0.77 | -27.99 | 37.91 |
| Toilet facility used by household |  |  |  |  |
| Ventilated pit latrine (vs. flush toilet) | -23.80 | 0.44 | -84.05 | 36.44 |
| Pit latrine with roof (vs. flush toilet) | -6.05 | 0.84 | -66.12 | 54.02 |
| Traditional pit latrine (vs. flush toilet) | -11.12 | 0.75 | -79.15 | 56.91 |
| Purchase special foods for children  (vs. no) | 2.48 | 0.83 | -19.69 | 24.65 |
| Mother’s body size preference for herself |  |  |  |  |
| Normal weight (vs. underweight) | 75.75 | 0.15 | -26.60 | 178.10 |
| Overweight (vs. underweight) | 81.69 | 0.12 | -20.06 | 183.44 |
| Obese (vs. underweight) | 83.22 | 0.11 | -19.89 | 186.34 |
| Mother’s perception of healthy body size for herself |  |  |  |  |
| Overweight (vs. normal weight) | 0.91 | 0.97 | -43.38 | 45.19 |
| Obese (vs. normal weight) | 0.35 | 0.99 | -45.07 | 45.76 |
| No child cough in last 2 weeks (vs. yes) | -13.27 | 0.15 | -31.49 | 4.96 |
| No mother diarrhea in last 2 weeks (vs. yes) | 15.20 | 0.35 | -16.47 | 46.87 |
| Total number of household assets | 12.15 | 0.09 | -1.80 | 26.11 |
| HFIAS score | -7.87 | 0.17 | -19.12 | 3.38 |
| Total amount spent on food for household | **12.34** | **0.04** | **0.76** | **23.92** |
| Total amount spent on special foods for children <5 years | **13.61** | **0.04** | **0.89** | **26.33** |
| Taste preference: dairy | 5.61 | 0.23 | -3.62 | 14.84 |

| **Table 49.** Predictors of oil/fat intake among mothers in the dry season. Model estimates from multivariate linear regression. P<0.05 are bold. | | | | |
| --- | --- | --- | --- | --- |
| **Variable** | **Coefficient** | **P-value** | **Lower 95% CI** | **Upper 95% CI** |
| Rural (vs. urban) | -3.42 | 0.22 | -8.92 | 2.08 |
| Mother secondary education or higher (vs. less) | 0.55 | 0.77 | -3.24 | 4.35 |
| Main source of drinking water |  |  |  |  |
| Well (vs. piped) | 3.03 | 0.35 | -3.38 | 9.43 |
| Borehole (vs. piped) | 3.87 | 0.19 | -1.95 | 9.69 |
| Toilet facility used by household |  |  |  |  |
| Ventilated pit latrine (vs. flush toilet) | 3.00 | 0.46 | -5.00 | 11.01 |
| Pit latrine with roof (vs. flush toilet) | 1.28 | 0.75 | -6.47 | 9.04 |
| Traditional pit latrine (vs. flush toilet) | -2.33 | 0.62 | -11.42 | 6.76 |
| Who purchases most food the family consumes |  |  |  |  |
| Husband/partner (vs. mother) | 3.74 | 0.11 | -0.88 | 8.35 |
| Both (vs. mother) | 5.33 | 0.08 | -0.59 | 11.25 |
| Other family member (vs. mother) | -0.54 | 0.88 | -7.34 | 6.25 |
| Purchase special foods for children  (vs. no) | 1.47 | 0.41 | -2.05 | 4.98 |
| Mother’s body size preference for herself |  |  |  |  |
| Normal weight (vs. underweight) | -8.71 | 0.28 | -24.42 | 7.01 |
| Overweight (vs. underweight) | -7.14 | 0.37 | -22.75 | 8.47 |
| Obese (vs. underweight) | -10.68 | 0.19 | -26.78 | 5.43 |
| Household size | -0.23 | 0.78 | -1.90 | 1.43 |
| Total number of household assets | **3.08** | **0.01** | **0.70** | **5.46** |
| HFIAS score | -1.13 | 0.24 | -3.00 | 0.74 |
| Total amount spent on food for household | -1.01 | 0.32 | -2.99 | 0.97 |

| **Table 50.** Predictors of oil/fat intake among mothers in the rainy season. Model estimates from multivariate linear regression. P<0.05 are bold. | | | | |
| --- | --- | --- | --- | --- |
| **Variable** | **Coefficient** | **P-value** | **Lower 95% CI** | **Upper 95% CI** |
| Rural (vs. urban) | -0.66 | 0.81 | -6.12 | 4.81 |
| Mother secondary education or higher (vs. less) |  |  |  |  |
| Yes | 1.75 | 0.33 | -1.82 | 5.32 |
| Main source of drinking water |  |  |  |  |
| Well (vs. piped) | -1.23 | 0.73 | -8.35 | 5.89 |
| Borehole (vs. piped) | -0.95 | 0.75 | -6.68 | 4.78 |
| Toilet facility used by household |  |  |  |  |
| Ventilated pit latrine (vs. flush toilet) | -1.78 | 0.74 | -12.23 | 8.67 |
| Pit latrine with roof (vs. flush toilet) | -2.05 | 0.70 | -12.58 | 8.47 |
| Traditional pit latrine (vs. flush toilet) | -2.13 | 0.72 | -13.97 | 9.72 |
| Mother’s body size preference for herself |  |  |  |  |
| Normal weight (vs. underweight) | -6.23 | 0.49 | -23.87 | 11.41 |
| Overweight (vs. underweight) | -1.23 | 0.89 | -18.79 | 16.33 |
| Obese (vs. underweight) | -3.48 | 0.70 | -21.27 | 14.31 |
| Mother’s perception of healthy body size for herself |  |  |  |  |
| Overweight (vs. normal weight) | 3.41 | 0.38 | -4.29 | 11.11 |
| Obese (vs. normal weight) | 2.34 | 0.56 | -5.55 | 10.24 |
| No mother cough in last 2 weeks (vs. yes) | 0.70 | 0.70 | -2.83 | 4.22 |
| Total number of household assets | 1.56 | 0.20 | -0.84 | 3.96 |
| HFIAS score | **-2.02** | **0.03** | **-3.84** | **-0.20** |
| Female autonomy | 0.81 | 0.31 | -0.77 | 2.40 |
| Total amount spent on food for household | 0.31 | 0.76 | -1.70 | 2.33 |
| How long it takes to get to nearest market/shop to purchase food | **-2.11** | **0.01** | **-3.72** | **-0.51** |
| Total amount spent on special foods for children <5 years | 1.31 | 0.16 | -0.53 | 3.15 |
| Taste preference: oil/fat | 1.27 | 0.12 | -0.33 | 2.88 |

| **Table 51.** Predictors of oil/fat intake among children in the dry season. Model estimates from multivariate linear regression. P<0.05 are bold. | | | | |
| --- | --- | --- | --- | --- |
| **Variable** | **Coefficient** | **P-value** | **Lower 95% CI** | **Upper 95% CI** |
| Dyad Type |  |  |  |  |
| Overweight mother, normal weight child (vs. both overweight) | 1.37 | 0.47 | -2.39 | 5.13 |
| Overweight child, normal weight mother (vs. both overweight) | 1.39 | 0.50 | -2.66 | 5.43 |
| Rural (vs. urban) | -1.71 | 0.49 | -6.61 | 3.18 |
| Mother secondary education or higher (vs. less) | -0.66 | 0.71 | -4.12 | 2.79 |
| Main source of drinking water |  |  |  |  |
| Well (vs. piped) | 2.47 | 0.40 | -3.30 | 8.24 |
| Borehole (vs. piped) | 4.08 | 0.13 | -1.15 | 9.31 |
| Toilet facility used by household |  |  |  |  |
| Ventilated pit latrine (vs. flush toilet) | **13.32** | **0.00** | **6.04** | **20.59** |
| Pit latrine with roof (vs. flush toilet) | 5.53 | 0.12 | -1.47 | 12.53 |
| Traditional pit latrine (vs. flush toilet) | 4.57 | 0.27 | -3.65 | 12.79 |
| Who purchases most food the family consumes |  |  |  |  |
| Husband/partner (vs. mother) | 2.57 | 0.24 | -1.76 | 6.89 |
| Both (vs. mother) | **7.49** | **0.01** | **2.11** | **12.87** |
| Other family member (vs. mother) | -0.17 | 0.96 | -7.06 | 6.71 |
| Other transport to buy food (vs. walk) | 6.24 | 0.07 | -0.57 | 13.05 |
| Purchase special foods for children  (vs. no) | 0.66 | 0.69 | -2.60 | 3.91 |
| No mother diarrhea in last 2 weeks (vs. yes) | **5.43** | **0.03** | **0.65** | **10.22** |
| Age of mother | 0.42 | 0.61 | -1.23 | 2.07 |
| Age of child | **2.64** | **0.00** | **1.04** | **4.25** |
| Total number of household assets | **2.51** | **0.02** | **0.35** | **4.67** |
| HFIAS score | -1.02 | 0.24 | -2.70 | 0.67 |
| Female autonomy | 0.35 | 0.70 | -1.41 | 2.10 |
| Total amount spent on food for household | 1.12 | 0.22 | -0.66 | 2.90 |
| How long it takes to get to nearest market/shop to purchase food | -1.56 | 0.05 | -3.14 | 0.01 |

| **Table 52.** Predictors of oil/fat intake among children in the rainy season. Model estimates from multivariate linear regression. P<0.05 are bold. | | | | |
| --- | --- | --- | --- | --- |
| **Variable** | **Coefficient** | **P-value** | **Lower 95% CI** | **Upper 95% CI** |
| Rural (vs. urban) | -3.77 | 0.16 | -9.05 | 1.51 |
| Mother secondary education or higher (vs. less) | 0.89 | 0.61 | -2.58 | 4.37 |
| Main source of drinking water |  |  |  |  |
| Well (vs. piped) | -0.73 | 0.83 | -7.60 | 6.14 |
| Borehole (vs. piped) | 0.86 | 0.76 | -4.67 | 6.40 |
| Toilet facility used by household |  |  |  |  |
| Ventilated pit latrine (vs. flush toilet) | 3.40 | 0.51 | -6.66 | 13.47 |
| Pit latrine with roof (vs. flush toilet) | 1.59 | 0.76 | -8.55 | 11.73 |
| Traditional pit latrine (vs. flush toilet) | 4.30 | 0.46 | -7.15 | 15.75 |
| Mother’s body size preference for herself |  |  |  |  |
| Normal weight (vs. underweight) | 2.51 | 0.77 | -14.48 | 19.51 |
| Overweight (vs. underweight) | 6.33 | 0.46 | -10.59 | 23.25 |
| Obese (vs. underweight) | 5.76 | 0.51 | -11.38 | 22.90 |
| Mother’s perception of healthy body size for herself |  |  |  |  |
| Overweight (vs. normal weight) | 0.73 | 0.85 | -6.69 | 8.15 |
| Obese (vs. normal weight) | -1.00 | 0.80 | -8.61 | 6.61 |
| No mother cough in last 2 weeks (vs. yes) | 2.48 | 0.15 | -0.93 | 5.89 |
| Total number of household assets | 1.90 | 0.11 | -0.43 | 4.22 |
| HFIAS score | **-1.85** | **0.04** | **-3.61** | **-0.09** |
| Female autonomy | 0.18 | 0.82 | -1.35 | 1.71 |
| Total amount spent on food for household | 0.08 | 0.94 | -1.87 | 2.03 |
| How long it takes to get to nearest market/shop to purchase food | -0.91 | 0.25 | -2.46 | 0.63 |
| Total amount spent on special foods for children <5 years | 0.22 | 0.81 | -1.56 | 1.99 |
| Taste preference: oil/fat | **2.26** | **0.01** | **0.71** | **3.81** |

| **Table 53.** Predictors of snacks intake among mothers in the dry season. Model estimates from multivariate linear regression. P<0.05 are bold. | | | | |
| --- | --- | --- | --- | --- |
| **Variable** | **Coefficient** | **P-value** | **Lower 95% CI** | **Upper 95% CI** |
| Rural (vs. urban) | -15.24 | 0.38 | -49.42 | 18.93 |
| Mother secondary education or higher (vs. less) | 12.14 | 0.31 | -11.35 | 35.64 |
| Main source of drinking water |  |  |  |  |
| Well (vs. piped) | 2.79 | 0.89 | -37.54 | 43.12 |
| Borehole (vs. piped) | 7.78 | 0.67 | -28.56 | 44.11 |
| Toilet facility used by household |  |  |  |  |
| Ventilated pit latrine (vs. flush toilet) | 20.10 | 0.43 | -29.79 | 69.98 |
| Pit latrine with roof (vs. flush toilet) | 2.30 | 0.93 | -45.85 | 50.45 |
| Traditional pit latrine (vs. flush toilet) | 1.73 | 0.95 | -55.10 | 58.56 |
| Who purchases most food the family consumes |  |  |  |  |
| Husband/partner (vs. mother) | -17.38 | 0.24 | -46.24 | 11.48 |
| Both (vs. mother) | 17.18 | 0.36 | -19.99 | 54.35 |
| Other family member (vs. mother) | 41.00 | 0.06 | -1.17 | 83.17 |
| Purchase special foods for children  (vs. no) | **68.80** | **0.00** | **25.47** | **112.14** |
| Total number of household assets | 1.73 | 0.82 | -13.27 | 16.73 |
| HFIAS score | **-15.14** | **0.01** | **-26.72** | **-3.55** |
| Total amount spent on food for household | -5.24 | 0.41 | -17.70 | 7.22 |
| Total amount spent on special foods for children <5 years | **25.58** | **0.02** | **5.00** | **46.16** |

| **Table 54.** Predictors of snacks intake among mothers in the rainy season. Model estimates from multivariate linear regression. P<0.05 are bold. | | | | |
| --- | --- | --- | --- | --- |
| **Variable** | **Coefficient** | **P-value** | **Lower 95% CI** | **Upper 95% CI** |
| Rural (vs. urban) | -23.63 | 0.16 | -56.29 | 9.02 |
| Mother secondary education or higher (vs. less) | 2.82 | 0.79 | -18.49 | 24.13 |
| Main source of drinking water |  |  |  |  |
| Well (vs. piped) | -9.22 | 0.67 | -51.79 | 33.34 |
| Borehole (vs. piped) | 5.20 | 0.77 | -29.49 | 39.90 |
| Toilet facility used by household |  |  |  |  |
| Ventilated pit latrine (vs. flush toilet) | 59.56 | 0.06 | -1.77 | 120.90 |
| Pit latrine with roof (vs. flush toilet) | 47.46 | 0.13 | -13.74 | 108.66 |
| Traditional pit latrine (vs. flush toilet) | 50.00 | 0.16 | -19.10 | 119.10 |
| Purchase special foods for children  (vs. no) | 5.58 | 0.63 | -16.93 | 28.09 |
| Age of mother | -6.50 | 0.20 | -16.45 | 3.45 |
| Total number of household assets | -3.84 | 0.60 | -18.27 | 10.60 |
| HFIAS score | -9.96 | 0.09 | -21.41 | 1.49 |
| Total amount spent on food for household | 0.46 | 0.94 | -11.72 | 12.65 |
| Total amount spent on special foods for children <5 years | **32.05** | **0.00** | **19.14** | **44.95** |
| Taste preference: snacks | 3.28 | 0.50 | -6.19 | 12.74 |

| **Table 55.** Predictors of snacks intake among children in the dry season. Model estimates from multivariate linear regression. P<0.05 are bold. | | | | |
| --- | --- | --- | --- | --- |
| **Variable** | **Coefficient** | **P-value** | **Lower 95% CI** | **Upper 95% CI** |
| Rural (vs. urban) | -27.25 | 0.05 | -54.61 | 0.11 |
| Mother secondary education or higher (vs. less) | 4.86 | 0.62 | -14.21 | 23.93 |
| Main source of drinking water |  |  |  |  |
| Well (vs. piped) | -5.83 | 0.72 | -38.01 | 26.35 |
| Borehole (vs. piped) | 16.21 | 0.28 | -12.98 | 45.40 |
| Toilet facility used by household |  |  |  |  |
| Ventilated pit latrine (vs. flush toilet) | -11.90 | 0.56 | -52.35 | 28.54 |
| Pit latrine with roof (vs. flush toilet) | -14.94 | 0.45 | -53.87 | 23.99 |
| Traditional pit latrine (vs. flush toilet) | -17.93 | 0.44 | -63.05 | 27.20 |
| Purchase special foods for children  (vs. no) | 25.73 | 0.15 | -9.16 | 60.62 |
| No child cough in last 2 weeks (vs. yes) | 4.55 | 0.60 | -12.58 | 21.69 |
| Age of child | **17.81** | **0.00** | **9.50** | **26.13** |
| Household size | -4.80 | 0.25 | -13.03 | 3.42 |
| Total number of household assets | 5.60 | 0.36 | -6.49 | 17.69 |
| HFIAS score | **-10.70** | **0.03** | **-20.02** | **-1.39** |
| Total amount spent on food for household | 1.05 | 0.84 | -9.11 | 11.21 |
| Total amount spent on special foods for children <5 years | 6.95 | 0.41 | -9.54 | 23.44 |

| **Table 56.** Predictors of snacks intake among children in the rainy season. Model estimates from multivariate linear regression. P<0.05 are bold. | | | | |
| --- | --- | --- | --- | --- |
| **Variable** | **Coefficient** | **P-value** | **Lower 95% CI** | **Upper 95% CI** |
| Kasungu district (vs. Lilongwe) | -6.80 | 0.51 | -26.89 | 13.29 |
| Rural (vs. urban) | **-42.58** | **0.02** | **-77.25** | **-7.92** |
| Mother secondary education or higher (vs. less) | 1.59 | 0.89 | -21.53 | 24.71 |
| Main source of drinking water |  |  |  |  |
| Well (vs. piped) | 24.91 | 0.28 | -20.34 | 70.16 |
| Borehole (vs. piped) | 17.49 | 0.35 | -19.06 | 54.04 |
| Toilet facility used by household |  |  |  |  |
| Ventilated pit latrine (vs. flush toilet) | 54.57 | 0.10 | -10.40 | 119.53 |
| Pit latrine with roof (vs. flush toilet) | 22.06 | 0.50 | -42.85 | 86.98 |
| Traditional pit latrine (vs. flush toilet) | 40.34 | 0.28 | -32.99 | 113.68 |
| Purchase special foods for children  (vs. no) | 4.87 | 0.69 | -19.48 | 29.21 |
| Mother’s perception of healthy body size for herself |  |  |  |  |
| Overweight (vs. normal weight) | -32.31 | 0.18 | -79.28 | 14.66 |
| Obese (vs. normal weight) | -0.13 | 1.00 | -47.50 | 47.23 |
| No mother cough in last 2 weeks (vs. yes) | 4.39 | 0.71 | -18.81 | 27.59 |
| Total number of household assets | 9.80 | 0.21 | -5.52 | 25.12 |
| HFIAS score | **-14.30** | **0.02** | **-26.52** | **-2.08** |
| Female autonomy | 3.95 | 0.45 | -6.35 | 14.25 |
| Total amount spent on food for household | -1.44 | 0.83 | -14.38 | 11.49 |
| Total amount spent on special foods for children <5 years | **22.15** | **0.00** | **8.34** | **35.97** |
| Taste preference: snacks | 8.32 | 0.11 | -1.93 | 18.57 |

| **Table 57.** Predictors of sweets intake among mothers in the dry season. Model estimates from multivariate linear regression. P<0.05 are bold. | | | | |
| --- | --- | --- | --- | --- |
| **Variable** | **Coefficient** | **P-value** | **Lower 95% CI** | **Upper 95% CI** |
| Rural (vs. urban) | -40.25 | 0.30 | -116.59 | 36.10 |
| Mother secondary education or higher (vs. less) | 26.51 | 0.32 | -25.89 | 78.91 |
| Main source of drinking water |  |  |  |  |
| Well (vs. piped) | 3.45 | 0.94 | -83.80 | 90.71 |
| Borehole (vs. piped) | 44.02 | 0.28 | -36.29 | 124.33 |
| Toilet facility used by household |  |  |  |  |
| Ventilated pit latrine (vs. flush toilet) | 34.16 | 0.55 | -76.73 | 145.04 |
| Pit latrine with roof (vs. flush toilet) | 24.43 | 0.65 | -82.53 | 131.39 |
| Traditional pit latrine (vs. flush toilet) | 19.41 | 0.76 | -105.13 | 143.95 |
| Who purchases most food the family consumes |  |  |  |  |
| Husband/partner (vs. mother) | -42.77 | 0.20 | -108.97 | 23.43 |
| Both (vs. mother) | -15.98 | 0.70 | -97.79 | 65.84 |
| Other family member (vs. mother) | -4.91 | 0.93 | -110.02 | 100.21 |
| Other transport to buy food (vs. walk) | **119.50** | **0.02** | **17.48** | **221.51** |
| Purchase special foods for children  (vs. no) | 88.94 | 0.07 | -7.31 | 185.18 |
| Mother’s perception of healthy body size for herself |  |  |  |  |
| Overweight (vs. normal weight) | 100.28 | 0.07 | -7.19 | 207.75 |
| Obese (vs. normal weight) | 82.17 | 0.17 | -35.85 | 200.19 |
| Mother’s perception of healthy body size for her child |  |  |  |  |
| Normal weight (vs. underweight) | **-520.02** | **0.01** | **-897.45** | **-142.60** |
| Overweight (vs. underweight) | **-453.22** | **0.02** | **-823.61** | **-82.82** |
| Obese (vs. underweight) | **-503.28** | **0.01** | **-874.85** | **-131.70** |
| No mother fever in last 2 weeks (vs. yes) | 1.46 | 0.97 | -69.23 | 72.14 |
| Total number of household assets | 20.90 | 0.22 | -12.72 | 54.52 |
| HFIAS score | -20.78 | 0.11 | -46.15 | 4.59 |
| Female autonomy | -1.82 | 0.89 | -27.38 | 23.73 |
| Total amount spent on food for household | 14.13 | 0.31 | -13.10 | 41.35 |
| Total amount spent on special foods for children <5 years | 39.67 | 0.09 | -5.62 | 84.95 |

| **Table 58.** Predictors of sweets intake among mothers in the rainy season. Model estimates from multivariate linear regression. P<0.05 are bold. | | | | |
| --- | --- | --- | --- | --- |
| **Variable** | **Coefficient** | **P-value** | **Lower 95% CI** | **Upper 95% CI** |
| Dyad Type |  |  |  |  |
| Overweight mother, normal weight child (vs. both overweight) | **-96.52** | **0.01** | **-167.64** | **-25.40** |
| Overweight child, normal weight mother (vs. both overweight) | **-104.33** | **0.01** | **-180.25** | **-28.41** |
| Rural (vs. urban) | -36.02 | 0.48 | -136.38 | 64.33 |
| Mother secondary education or higher (vs. less) | 60.68 | 0.07 | -5.66 | 127.01 |
| Main source of drinking water |  |  |  |  |
| Well (vs. piped) | 10.16 | 0.88 | -121.72 | 142.04 |
| Borehole (vs. piped) | 2.32 | 0.97 | -104.12 | 108.76 |
| Toilet facility used by household |  |  |  |  |
| Ventilated pit latrine (vs. flush toilet) | -82.85 | 0.39 | -273.33 | 107.63 |
| Pit latrine with roof (vs. flush toilet) | 19.63 | 0.84 | -170.02 | 209.28 |
| Traditional pit latrine (vs. flush toilet) | 19.93 | 0.86 | -194.37 | 234.23 |
| Purchase special foods for children  (vs. no) | 2.12 | 0.95 | -67.13 | 71.37 |
| Mother’s perception of healthy body size for herself |  |  |  |  |
| Overweight (vs. normal weight) | 13.46 | 0.85 | -124.73 | 151.65 |
| Obese (vs. normal weight) | -35.66 | 0.61 | -173.73 | 102.40 |
| Total number of household assets | **64.44** | **0.00** | **20.22** | **108.66** |
| HFIAS score | -12.70 | 0.49 | -48.64 | 23.23 |
| Total amount spent on food for household | -8.33 | 0.66 | -45.51 | 28.85 |
| Total amount spent on special foods for children <5 years | -14.00 | 0.49 | -53.66 | 25.67 |
| Taste preference: sweets | 7.81 | 0.60 | -21.80 | 37.42 |

| **Table 59.** Predictors of sweets intake among children in the dry season. Model estimates from multivariate linear regression. P<0.05 are bold. | | | | |
| --- | --- | --- | --- | --- |
| **Variable** | **Coefficient** | **P-value** | **Lower 95% CI** | **Upper 95% CI** |
| Dyad Type |  |  |  |  |
| Overweight mother, normal weight child (vs. both overweight) | -28.67 | 0.16 | -68.90 | 11.56 |
| Overweight child, normal weight mother (vs. both overweight) | -10.84 | 0.62 | -54.19 | 32.50 |
| Rural (vs. urban) | -4.28 | 0.87 | -57.49 | 48.92 |
| Mother secondary education or higher (vs. less) | **51.57** | **0.01** | **15.16** | **87.98** |
| Main source of drinking water |  |  |  |  |
| Well (vs. piped) | -21.82 | 0.48 | -82.77 | 39.13 |
| Borehole (vs. piped) | 4.57 | 0.87 | -51.38 | 60.51 |
| Toilet facility used by household |  |  |  |  |
| Ventilated pit latrine (vs. flush toilet) | **-79.38** | **0.04** | **-154.92** | **-3.85** |
| Pit latrine with roof (vs. flush toilet) | -60.91 | 0.10 | -133.15 | 11.33 |
| Traditional pit latrine (vs. flush toilet) | -75.36 | 0.08 | -159.18 | 8.47 |
| Purchase special foods for children  (vs. no) | **109.76** | **0.00** | **43.07** | **176.44** |
| Mother’s perception of healthy body size for herself |  |  |  |  |
| Overweight (vs. normal weight) | **100.19** | **0.01** | **25.03** | **175.36** |
| Obese (vs. normal weight) | **121.41** | **0.00** | **38.91** | **203.92** |
| Mother’s perception of healthy body size for her child |  |  |  |  |
| Normal weight (vs. underweight) | -56.26 | 0.68 | -322.64 | 210.12 |
| Overweight (vs. underweight) | -120.64 | 0.36 | -382.18 | 140.90 |
| Obese (vs. underweight) | -147.71 | 0.27 | -410.50 | 115.08 |
| No child diarrhea in last 2 weeks (vs. yes) | 12.59 | 0.50 | -24.28 | 49.47 |
| No mother fever in last 2 weeks (vs. yes) | 4.40 | 0.86 | -45.91 | 54.71 |
| Age of mother | 3.47 | 0.69 | -13.72 | 20.67 |
| Age of child | **40.35** | **0.00** | **22.83** | **57.88** |
| Total number of household assets | 18.99 | 0.11 | -4.49 | 42.47 |
| HFIAS score | -5.08 | 0.58 | -23.01 | 12.85 |
| Female autonomy | 7.85 | 0.35 | -8.71 | 24.41 |
| Total amount spent on food for household | 14.36 | 0.14 | -4.79 | 33.50 |
| Total amount spent on special foods for children <5 years | **39.78** | **0.01** | **8.24** | **71.32** |

| **Table 60.** Predictors of sweets intake among children in the dry season. Model estimates from multivariate linear regression. P<0.05 are bold. | | | | |
| --- | --- | --- | --- | --- |
| **Variable** | **Coefficient** | **P-value** | **Lower 95% CI** | **Upper 95% CI** |
| Dyad Type |  |  |  |  |
| Overweight mother, normal weight child (vs. both overweight) | 26.23 | 0.25 | -18.93 | 71.39 |
| Overweight child, normal weight mother (vs. both overweight) | -1.64 | 0.95 | -49.78 | 46.50 |
| Rural (vs. urban) | -27.18 | 0.39 | -89.59 | 35.24 |
| Mother secondary education or higher (vs. less) | **49.29** | **0.02** | **7.44** | **91.14** |
| Main source of drinking water |  |  |  |  |
| Well (vs. piped) | 28.55 | 0.50 | -54.29 | 111.39 |
| Borehole (vs. piped) | 19.89 | 0.56 | -47.16 | 86.95 |
| Toilet facility used by household |  |  |  |  |
| Ventilated pit latrine (vs. flush toilet) | -93.21 | 0.12 | -211.47 | 25.06 |
| Pit latrine with roof (vs. flush toilet) | **-120.22** | **0.05** | **-237.76** | **-2.68** |
| Traditional pit latrine (vs. flush toilet) | -119.47 | 0.08 | -253.55 | 14.60 |
| Purchase special foods for children  (vs. no) | **43.53** | **0.05** | **0.35** | **86.72** |
| Mother’s perception of healthy body size for herself |  |  |  |  |
| Overweight (vs. normal weight) | **-108.53** | **0.02** | **-195.41** | **-21.64** |
| Obese (vs. normal weight) | **-86.44** | **0.05** | **-172.30** | **-0.59** |
| Age of child | 16.46 | 0.12 | -4.09 | 37.01 |
| Total number of household assets | 14.10 | 0.31 | -13.23 | 41.44 |
| HFIAS score | -13.07 | 0.25 | -35.33 | 9.19 |
| Total amount spent on food for household | 10.85 | 0.36 | -12.29 | 33.99 |
| Total amount spent on special foods for children <5 years | 23.11 | 0.07 | -1.51 | 47.72 |
